# Identification of single nucleotide polymorphisms in sheep Mx genes: A premature stop codon abolishes Mx2 protein expression but did not affect fertility and early animal development

**DOI:** 10.1371/journal.pone.0337457

**Published:** 2026-02-11

**Authors:** Cindy Eunhee Lee, Nahideh Moradi, Brad Hine, Nicholas M. Andronicos, Jody McNally, Jian-Wei Liu, Tanja Strive, Ina Smith, Peter W. Hunt, Michael Frese

**Affiliations:** 1 Faculty of Science and Technology, University of Canberra, Bruce, Australian Capital Territory, Australia; 2 CSIRO Health and Biosecurity, Canberra, Australian Capital Territory, Australia; 3 CSIRO Agriculture and Food, F.D. M^c^Master Laboratory, Armidale, New South Wales, Australia; 4 School of Science and Technology, University of New England, Armidale, New South Wales, Australia; 5 CSIRO Environment, Canberra, Australian Capital Territory, Australia; ICAR-Indian Veterinary Research Institute: Indian Veterinary Research Institute, INDIA

## Abstract

Mx proteins are interferon-induced GTPases that inhibit a wide range of viruses. The loss of functional Mx genes in mice and other model species is associated with inferior innate immune responses and increased virus susceptibility. Here, we describe genetic variations in the Mx genes of sheep (*Ovis aries*). More than 700 single nucleotide polymorphisms within or adjacent to *MX1* and *MX2* were identified by analysing whole genome sequence data from 68 sheep, representing 43 breeds from 19 countries. Amongst those are two biologically significant variations in the ovine *MX2* gene: a guanosine-to-adenosine transition that generates a stop at codon 166 (c.497G > A; p.W166*) and a single nucleotide deletion in codon 329 that creates a frameshift and a premature stop of translation eight codons later (c.985del; p.Q329Sfs7*). A subsequent genotyping of Australian Merino sheep identified animals with a stop codon at position 166 in two research flocks that have been kept as closed flocks since the 1970s. Immunoblotting, immunofluorescence and mass spectrometry assays show that animals homozygous for the defect do not express detectable amounts of Mx2 proteins, and quantitative PCR suggests that the premature stop codon destabilises Mx2 mRNA. Furthermore, we found that Mx2-negative ewes and rams are fertile and that Mx2-negative lambs are indistinguishable from heterozygotes and wild-type animals in appearance, birth weight and growth rate.

## Introduction

Mx (myxovirus resistance) proteins belong to a family of dynamin-like large GTPases with intrinsic antiviral activities [1]. In vertebrates, the expression of Mx proteins is strictly regulated by interferons (IFNs), i.e., from low base levels, expression quickly increases in response to type I and type III but not type II IFNs [2]. With the exception of stem cells, most vertebrate cell types rely on Mx proteins for the establishment of an IFN-induced antiviral state, a key element of the innate immune response [3].

All Mx proteins contain a conserved N-terminal GTPase domain, a ‘stalk’ with the middle domain containing the L4 loop (involved in interactions with viral ribonucleoproteins), and several bundle-signalling elements (BSEs). Furthermore, most Mx proteins contain a leucine zipper, and some have a nuclear localisation signal (NLS). The antiviral activity of Mx proteins relies on direct interactions with viral components, a process that is dependent on the formation of highly structured oligomers and, in most cases, GTPase activity. Mx proteins inhibit the replication of a wide range of RNA and DNA viruses; the interaction with viral proteins and/or ribonucleoprotein complexes is rather promiscuous and underlying principles are not well understood (for a more detailed description of these proteins and comprehensive listings of Mx-sensitive viruses, see [2,4,5]). Most mammalian species possess two or more functional Mx genes. The importance of having functional Mx genes was first recognised in laboratory mice that carry non-functional Mx alleles [6]. *MX1*-negative mice (and cells that were derived from these mice) are highly susceptible to the *Influenza A virus* and other orthomyxoviruses, whereas *MX1*-positive animals (and cells) are highly virus resistant [7–11].

Sheep (*Ovis aries*) possess two Mx genes (*MX1* and *MX2*) that are located in close proximity on chromosome 1 [12]. Notably, the first and most subsequent studies on ovine Mx proteins were not conducted by virologists but rather by researchers interested in reproduction [13–22]. Sheep and other ruminants rely on IFN-τ, a type I IFN, as the primary signalling molecule for maternal recognition of pregnancy. The production of IFN-τ extends not only the life span of the corpus luteum but also activates numerous genes, such as *MX1* and *MX2* [23,24]. Thus, it is not surprising that the spatial and temporal correlation of Mx expression with key steps in early pregnancy has sparked the interest of reproductive biologists and led to speculations that Mx proteins play a role in the reproduction of ruminants [18,20,25,26].

While most non-rodent Mx1 proteins (including the human ortholog MxA) have a broad antiviral activity and inhibit a range of RNA and DNA viruses, Mx2 proteins (including the human MxB protein) were thought to lack antiviral activity [27,28]. This notion changed with the observation that MxB inhibits the replication of the *Human immunodeficiency virus* [29]. Since this discovery, more viruses were found to be susceptible to MxB, such as the *Human hepatitis B virus* [30], *Hepatitis C virus* [31] and several herpesviruses [32,33].

When Busnadiego and coworkers [34] compared the intracellular localisation and anti-retroviral activity of MxB to those of ovine Mx2 and other non-primate Mx2 orthologs using a tetracycline-inducible retroviral expression system, they found that non-primate Mx2 proteins do not associate with nuclear pores and do not inhibit the replication of retroviruses. Other findings, however, suggest that ovine Mx proteins possess antiviral activity against retroviruses. According to Larruskain and co-workers [35], single nucleotide polymorphisms (SNPs) in *MX1* are associated with an increased susceptibility to ovine pulmonary adenocarcinoma, a ‘contagious’ lung cancer that is caused by the *Jaagsiekte sheep retrovirus*. Given the proximity of *MX1* and *MX2*, any SNP in or near *MX1* is close to *MX2*. Therefore, further research is needed to fully understand the relationship of these SNPs with the observed susceptibility to virus-associated diseases.

Here, we report the findings of a ‘data mining’ exercise in which we analysed data from 68 individual sheep, representing 43 breeds from 19 countries [36]. The investigation revealed the existence of two non-functional *MX2* alleles with premature stop codons (W166* and Q329Sfs7*). Furthermore, we found the W166* stop codon in two out of four Merino flocks at the CSIRO F.D. McMaster Research Laboratory in Armidale, Australia. Biochemistry assays determined that sheep homozygous for the W166* stop codon do not express a detectable amount of the Mx2 protein. Finally, we will discuss potential implications of non-functional Mx alleles in production animals with respect to antiviral resistance and animal reproduction.

## Results

### Localisation and organisation of ovine Mx genes

**Fig 1:**
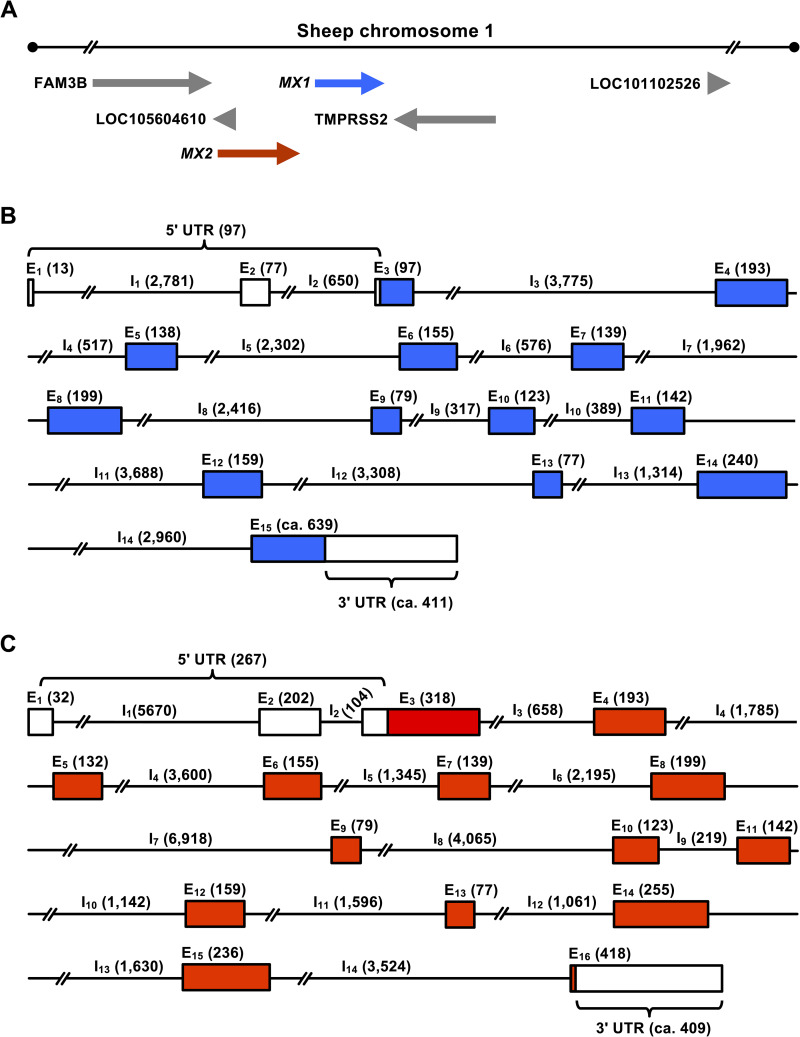
Localisation and organisation of ovine Mx genes. (A) Position of *MX1* and *MX2* on the chromosome 1. (B, C) Gene organisation of *MX1* and *MX2*, respectively. Boxes, exons (E); black lines, introns (I); blue, *MX1* coding sequences; red, *MX2* coding sequences; white, untranslated regions (UTRs); numbers in brackets, number of nucleotides in a particular UTR, intron or exon.

Both ovine *MX1* and *MX2* are located closely together on the distal part of chromosome 1q; they are flanked by *FAM3B* and *TMPRSS2* like in other mammals such as humans, dogs and pigs (Fig 1A). As only about 8,000 bp separate *MX1* and *MX2*, both genes are usually inherited together. By comparing ovine and bovine *MX1* sequences [37], we identified splice donor and acceptor sites in the ovine *MX1* gene and constructed a map with 15 exons of which all but the first two contain coding information (Fig 1B). To map the ovine *MX2* gene, we used published ovine Mx2 cDNA sequences (GenBank accession no. AY859475 and KF925355), the sheep genome sequence [12], and our own sequence data. Our analysis revealed that the *MX2* gene contains 16 exons (one more than previously thought; GenBank accession no. NC_056054) of which all but the first two contain coding sequences (Fig 1C).

Next, we searched the promoter region of both ovine Mx genes up to approximately 1,000 nucleotides (nts) upstream of the first nucleotide in exon 1 for transcription factor binding sites, especially for sequences that indicate the presence of IFN-regulatory response elements (S1 Fig). Our analysis was guided by previous studies on the promoter region of the ovine *MX1* [17] and the two bovine Mx genes [38,39]. As expected, we found that the promoters of the ovine Mx genes contain a minimum of two putative ISREs, several GC-rich motifs, GC boxes and putative binding sites for the activator protein 1 (AP-1), the ‘specificity protein’ (SP1), the CCAAT/enhancer-binding protein beta (CEBPB) and the nuclear factor kappa-light-chain-enhancer of activated B cells (NF-κB). Furthermore, we found a typical TATA motif upstream of the presumed transcription start site in ovine *MX2* (no such motif exists in ovine *MX1*) (S1 Fig). While the presence of at least one ISRE is a hallmark of all Mx genes in vertebrates, the importance of other motifs is less clear. For example, a TATA motif was found in the Mx genes of mice [40] and the water buffalo *MX2* gene [38] but not in the Mx genes of other mammalian species [e.g., 37,41,42]. Whether the presence of a TATA motif in the promoter of the ovine *MX2* changes its expression pattern compared to that of the ovine *MX1* gene is presently unknown; functional studies are required to answer the question.

**Fig 2:**
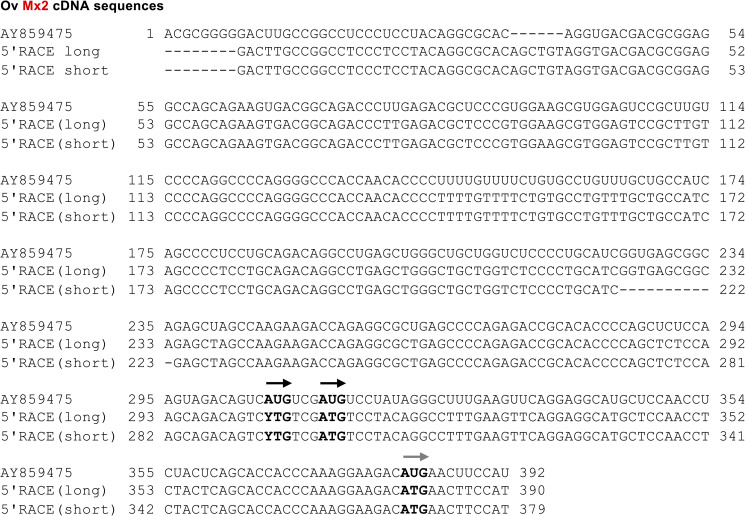
Alignment of different ovine Mx2 cDNA sequences. Top sequence, GenBank reference mRNA sequence for ovine Mx2 (AY869475); middle and bottom, cDNA sequences resulting from our 5’RACE sequencing. Potential start codons are printed in bold. The position of start codons for long and short Mx2 variants are indicated by black and grey arrows, respectively. Note that further sequence comparisons suggest that the ATG codon at position 306–8 (AY859475) is likely used to start the synthesis of ovine Mx2 proteins. The letter Y stands for a G or an A (sequencing data suggest that the sample used for the 5’RACE sequencing was obtained from a heterozygous animal).

For ovine Mx1 transcripts, the sequence of the 5’ UTR has previously been determined using 5’ RACE [17], but the 5’ sequence of ovine Mx2 mRNAs has only been predicted by bioinformatics tools. Therefore, we conducted a 5’ RACE to determine the true 5’ sequence of the ovine Mx2 mRNAs, which revealed two mRNA populations of different length (297 and 303 nucleotides long) but of otherwise identical sequence (Fig 2). When we compared the 5’ RACE sequences to the reference mRNA sequence (GenBank accession no. AY869475), we found that the reference sequence has eight additional nts at its 5’ end but lacks a stretch of six nts further downstream (nt 31–36 in Fig 2). Interestingly, when we compared the mRNA sequences to a genomic reference sequence (ARS-UI_Ramb_v3.0; GenBank accession no. GCA_016772045.2), we found that the six additional nts aligned to a sequence in chromosome 1 (Chr 1:262,303,068–73), adjacent to the postulated start of transcription (GenBank accession no. AY859475; Chr 1:262303074). However, the genome reference sequence does not contain the first 30 nts of the 5’ UTR, but further sequence comparisons revealed that the 5’ UTR has a match in the updated reference genome sequence Oar_v4.0 (GCA_000298735.2), i.e., the 32 nts of the 5’ end perfectly matched chromosome 1:259,515,555–86. The next 202 nts also have a 100% match in the reference sequence, located 5,789 nucleotides downstream of the first match (Chr 1:259,521,374–552), and the remaining 5’UTR sequence was found 102 nts further downstream (Chr 1: 259,521,676–748). These findings suggest that our 5’ RACE amplicons are copies of genuine Mx2 mRNAs and that three exons contribute to the 5’ UTR of ovine Mx2 mRNAs.

The ovine *MX2* reference sequence Oar_v4.0 (GCA_000298735.2) contains three ATGs that could be used as start codons (*O. aries* Gene ID: 780441); however, our 5’ RACE sequences revealed the existence of a heterozygous SNP (G/A) at the position of the first ATG, suggesting that the *MX2* gene contains either two or three in-frame ATG codons that may be used to start protein synthesis (Fig 2). Our mass spectrometry analysis detected two different peptides with sequences upstream of the third methionine, suggesting that one of the first two start codons were used (see below for more details). In addition, sequence comparisons of orthologs of the ovine *MX2* gene show that the first methionine residue is conserved across many mammalian species, including humans, cattle and dogs. Taken together, our results support the notion that the first ATG is likely the start codon of ovine *MX2* gene.

### Allelic variation in ovine *MX1* and *MX2*

To determine the degree of variation in ovine Mx genes, we obtained published whole genome sequence data of 68 sheep representing 43 breeds from 19 countries that had been assembled by the International Sheep Genomic Consortium [36,43] and analysed the data for SNPs and insertion-deletion events (INDELs). For the *MX1* gene, sequence information from 67 animals was available; we analysed a 70,218-bp region that included exons 2–15 (26,174 bp), a 641-bp sequence upstream of exon 2 and a 40,498-bp sequence downstream of exon 15. For the *MX2* gene, sequences were available from all 68 animals, and we analysed a 32,696-bp region containing 4,394 bp of the 5’ non-coding sequence, exons 3–14 (2,823 bp) and introns 4–13 (18,957 bp); exon 15, exon 16 and the 3’ untranslated sequences were not included in the analysis because of errors in the annotation of the genome at the time. It is important to note that our analysis aimed to assess the degree of genetic variation across the world’s sheep breeds; the study was not designed to provide an accurate assessment of allelic frequencies within breeds.

Our analysis revealed that SNPs were the most common type of genetic variation with a ratio of approx. 70% and 30% for transitional and translational substitutions, respectively (Table 1). As expected, SNPs (and other sequence variations) were less common in exon than in intron sequences (11 vs 258 SNPs for *MX1* and 16 vs 385 SNPs for *MX2*). When we analysed the differences in the frequency and nature of these (and other) sequence variations between the two ovine Mx genes, we found that variations that change the amino acid sequence of the gene product were also more prevalent in *MX2* (6 changes) than *MX1* (3 changes). Furthermore, we found that 37 out of the 68 sheep carried an A-to-G transition at the first nucleotide of the start codon of *MX2* (Fig 1C), which leads to a change from methionine in the reference state to the alternative valine at codon 1 (M1V). Thirteen animals were homozygous for the alternative allele, and 24 animals were heterozygous for the A nucleotide. The impact of this and other non-synonymous SNPs was analysed using the bioinformatics tools SIFT and PolyPhen-2, but the analysis did not produce conclusive results (S1 Table). We therefore postpone the discussion on the impact of individual amino acid changes until experimental data on the antiviral activity of Mx2 variants becomes available.

**Table 1:** SNPs and other variations in ovine *MX1* and *MX2* coding sequences.

Gene	Position^a^	Change	Type	Reads (variant/total)	f(alt)	Animal no. (variant/total)	Codon	Change
***MX1***	16	C > T	SNP (Ts)	166/878	0.19	18/57	Ser	no
	17	G > A	SNP (Ts)	136/876	0.16	12/57	Gly	Ser
	30	T > C	SNP (Ts)	796/801	0.99	52/52	Met	Thr
	80	T > C	SNP (Ts)	200/569	0.35	24/40	Ser	no
	125	G > A	SNP (Ts)	281/741	0.38	30/51	Glu	no
	288	C > T	SNP (Ts)	142/692	0.21	18/46	Asp	no
	323	C > G	SNP (Tv)	104/681	0.15	14/47	Thr	no
	390	C > T	SNP (Ts)	25/629	0.04	4/44	Leu	no
	452	A > G	SNP (Ts)	7/700	0.01	2/51	Ile	Val
	575	G > A	SNP (Ts)	26/735	0.04	3/49	Ala	no
	579	A > G	SNP (Ts)	74/411	0.18	8/32	Glu	no
***MX2***	1	A > G	SNP (Ts)	254/551	0.46	27/39	Met	Val
	28	T > C	SNP (Ts)	557/585	0.95	40/40	Phe	no
	64	A > C	SNP (Tv)	42/612	0.07	4/43	Asn	His
	85	C > T	SNP (Ts)	318/674	0.47	28/43	Pro	Ser
	**166** ^**b**^	**G > A**	**SNP (Ts)**	**79/627**	**0.13**	**9/44**	**Trp**	**STOP**
	175	G > A	SNP (Ts)	282/619	0.46	32/46	Val	no
	**329** ^**b**^	**C**	**CNV1**	**14/689**	**0.02**	**3/49**	**Gln**	**Frameshift** ^**c**^
	376	G > A	SNP (Ts)	54/641	0.08	7/44	Glu	no
	378	T > C	SNP (Ts)	58/672	0.09	8/47	Ile	no
	422	T > C	SNP (Ts)	140/527	0.27	19/38	Ile	no
	561	G > A	SNP (Ts)	582/735	0.79	48/50	Ala	no
	584	C > T	SNP (Ts)	56/760	0.07	7/51	Tyr	no
	598	C > T	SNP (Ts)	108/743	0.15	15/51	Ser	no
	608	A > G	SNP (Ts)	580/688	0.84	44/46	Ser	no
	616	G > A	SNP (Ts)	192/728	0.26	21/49	Gln	no
	697	C > A	SNP (Tv)	621/781	0.8	50/53	Arg	no
	713	G > A	SNP (Ts)	32/727	0.04	4/49	Gly	Ser

^a^ Codon number in which the allelic variation was observed. ^b^ Variations that significantly shorten the ORF. ^c^ Frameshift causing the following changes to the amino acid sequence: Gln.Gln.Asp.Ile.Thr.Asn.Lys.Leu to Ser.Arg.Ile.Ser.Pro.Thr.Ser.STOP. SNPs that lead to a premature termination of the ORF are highlighted in pink.

**Fig 3:**
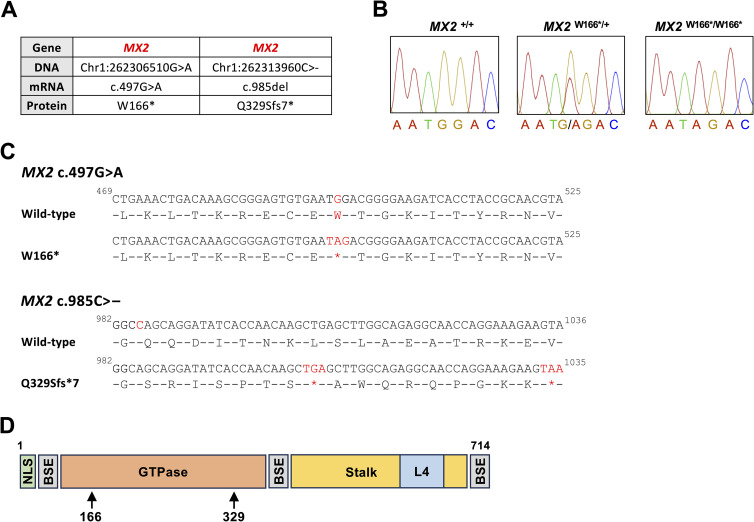
Nonfunctional ovine *MX2* alleles. (A) Two SNPs on chromosome 1 that generate premature stop codons in *MX2* (numbers refer to the positions in the sheep reference genome; GenBank accession no. NC_056054). (B) Sanger sequencing chromatograms demonstrating the presence of the W166* SNP in heterozygous (middle) and homozygous (right) Australian Merino sheep. (C) Predicted changes to the translation of Mx2 mRNAs in animals with the W166* SNP (top) and the Q329Sfs7* SNP (bottom). (D) Schematic representation of the ovine Mx2 protein (714 aa). Coloured boxes indicate the position of protein domains and other structural elements. NLS, nuclear localisation signal (green); BSE, bundle-signalling element (grey); GTPase, GTPase domain (orange); and stalk, stalk domain (yellow) with L4 loop (blue). The position of the ORF destroying SNPs W166* and Q329Sfs7* are indicated using arrows.

Notably, two of the observed variations in *MX2* result in premature STOP codons (Fig 3). A SNP in codon 166 changes a tryptophan codon (UGG) into the amber stop codon (UAG) (W166*). From the 68 animals investigated, this variation was observed in 13 animals (ten heterozygotes and three homozygotes) from 11 breeds (Cheviot, Dollgellau, Welsh Mountain, Finnsheep, Gulf Coast Native, Morada Nova, Santa Inês, Scottish Blackface, Swiss Mirror, Swiss White Alpine, Texel, Valais Blacknose) (Table 2); homozygous animals were found in Valais Blacknose and Texel, suggesting that at least some breeds have a high frequency for the non-functional *MX2* allele. The other allele that is likely nonfunctional contains an INDEL variation. A single nucleotide deletion in codon 329 likely causes a frameshift and generates an amber stop codon (Q329Sfs7*) in the middle of the original ORF. Taken together, our analysis suggests that animals across a wide range of breeds carry nonfunctional *MX2* alleles.

**Table 2:** Sheep breeds with non-functional *MX2* alleles.

Breeds	Genotype		Quantity^a^
Santa Inês, Swiss White Alpine, Finnsheep, Cheviot, Gulf Coast Native	W166*	heterozygote	11
Valais Blacknose, Texel		homozygote	2
Ethiopian Menz, Awassi, Sakiz	Q329Sfs7*	heterozygote	3

a Number of animals in which the SNP was found.

### Australian Merino sheep with a non-functional *MX2* allele

Genome sequencing revealed that several breeds of sheep carry *MX2* alleles with the premature stop codon W166* (Table 1). This finding prompted us to screen Australian sheep for the presence of this SNP. A first screening was carried out at the F.D. McMaster Research Station in Armidale (New South Wales) where four closed flocks of Australian Merinos are kept. Two of the flocks, referred to as HSFS and HSFR [44], were derived from a fine wool Merino strain; the other two flocks, referred to as TSFS and TSFR [45], were derived from the medium wool (Peppin) strain (for a description of the parental strain, see [46]). All four flocks have been maintained as closed flocks since the 1970s, which makes them a valuable resource for evaluating the long-term viability of animals carrying non-functional genes. These flocks were maintained with 80–100 females for each and four sire lines until selection was ceased in 2018.

Initially, 20 lambs from each flock (five lambs from each of the four sire groups) were sampled to obtain DNA for PCR and sequencing. In these animals, we found three transition SNPs in exon 5, the change that creates a stop at codon 166 (W166*), a missense change, ACG to GCG at codon 167 (p.T167A; c.499A > G), and a synonymous change, GTG to GTA at codon 175 (p.V175V; c.525G > A). Genotypes were determined by analysing chromatograms with animals classified as either homozygotes or heterozygotes (see example in Fig 3B), and allele frequencies were calculated for each of the four flocks (Table 3). In all cases, the results conformed to Hardy-Weinberg equilibrium expectations for a gene that is not under strong selection (p > 0.05). The frequency of the alternative allele of W166* was significantly higher in HSFR compared to HSFS sheep, and this allele was not present in the TSF animals sampled. The reference allele for V175V was more frequent in HSFR compared to HSFS animals, and its frequency was even higher in the TSF flocks. Allele frequencies did not differ significantly between any of the flocks for T167A. Sequencing additional animals with known pedigree revealed that some sires were heterozygotes, some were likely homozygotes and others had an ambiguous genotype. The analysis proved the existence of the alternate allele at W166* in two HSFR sires, and of the alternate allele at V175V in two HSFR and two HSFS sires. Given the progeny genotypes, it is also likely that one HSFS sire and all TSF sires are homozygotes for the reference allele at W166*; seven of the 16 sires were homozygotes for the reference allele at T167A, and four of the 16 sires were homozygotes for the reference allele at V175V. For three of the sixteen sire groups, all progenies had unambiguous haplotypes across the three SNPs, and these three sires, one from TSFR, and two from TSFS, were homozygotes for the reference alleles at W166* and T167A, and the alternate allele at V175V. The genotype of one of the HSFR sires was uncertain, given the progeny results for W166* and V175V. Samples were obtained from this sire (2012A0020) and 12 additional progenies. When the genotype data from all 17 progeny were considered, the sire was likely a homozygote for the alternative allele at W166*, a homozygote for the reference allele at T167A, and a heterozygote at V175V (Table 2). The analysis of the sire’s genotype is consistent with the genotype inferred from progeny results.

**Table 3:** Frequency of nonfunctional *MX2* alleles in Australian Merinos.

Flock^a^	W166*			T167A			V175V		
	TGG (Trp)	TGA (STOP)	n	GCG (Ala)	ACG (Thr)	n	GTG (Val)	GTA (Val)	n
HSFR	0.5	0.5^c^	33	0.1	0.9^b^	20	0.625	0.375^b^	20
HSFS	0.75	0.25^d^	20	0.05	0.95^b^	20	0.4	0.6^b^	20
TSFR	1	0^e^	20	0.1	0.9^b^	20	0.075	0.925^c^	20
TSFS	1	0^e^	20	0.05	0.95^b^	20	0.05	0.95^c^	2

Only SNPs in exon 5 of the *MX2* gene are shown. ^a^ HSFS and HSFR flocks were derived from a fine wool Merino strain; TSFS and TSFR were derived from the medium wool (Peppin) strain. All four flocks have been maintained as closed flocks since the 1970s. ^b, c, d, e^ Designate frequencies that are significantly different between flocks (letters in the reference allele column only, but these also apply for the alternate alleles). Significance groups are only valid within columns; “n” is the number of animals genotyped. P values range from 0.003 (TSFR vs HSFS for V175V) to 0 (TSFR vs HSFR for W166*). There are no significant differences in allele frequency for T167A as indicated by the superscripts.

Haplotypes observed did not include AGA or AGG, which is not surprising given the low likelihood of chromosome recombination within the 2-bp gap between the second position in codon 166 and the first in codon 167. It is remarkable, however, that there appears to have been a crossover in the 28-bp interval between the SNP at codon 166 and that at codon 175 as both AAG and AAA haplotypes were observed multiple times, and both GGA and GGG were observed once. The frequency of the null allele (W166*) and the presence of both homozygous and heterozygous individuals in the HSFR and HSFS flocks indicates that a nonfunctional *MX2* allele is not strongly deleterious; otherwise, natural selection would have eliminated the allele in more than twenty generations since the flocks have been kept genetically isolated.

### Functional *MX2* alleles are not required for successful breeding and early development

Additional breeding was conducted to reveal phenotypic effects of W166* (S2 Table). To this end, the null homozygote ram (2012A0020), and a heterozygous ram (2012A0063) were mated with twenty ewes each. In both cases, successful reproduction was observed, with no significant difference between the sire groups in the proportion of ewes conceiving (p = 0.42) or producing live lambs (p = 0.42), or between the number of live lambs produced (p = 0.11). Of the ewes, five were homozygous for the alternate allele at W166*, 12 were heterozygous, and 11 had the reference allele. Amongst the three genotypes, there also was no significant difference in conception or birth rate. A total of 52 lambs were born, of which 31 were genotyped for W166*, there were ten null alternate allele homozygotes for W166*, 14 heterozygotes, and seven wild-type. Lambs without functional *MX2* alleles showed no obvious difference in appearance to heterozygous or wild-type animals. The lambs were weighed at birth and when they were marked (tail docking and castration of males; 10–40 days later). Birth weights were not statistically different between lamb, dam and sire genotypes (S2 Table). We also analysed the weight at marking and the rate of live weight gain between birth and marking. Again, we found no statistical difference between different *MX2* genotypes (S2 Table). Taken together, the presence of the null alternate allele for W166* in either the homozygous or heterozygous state had little or no effect on the ability of rams or ewes to reproduce and on viability, birth weight and the initial growth of lambs. Future studies, however, may reveal a beneficial effect of functional *MX2* alleles on animal health and development, especially when ewes and/or lambs are challenged by viruses.

### Mx2 expression in sheep fibroblasts

**Fig 4:**
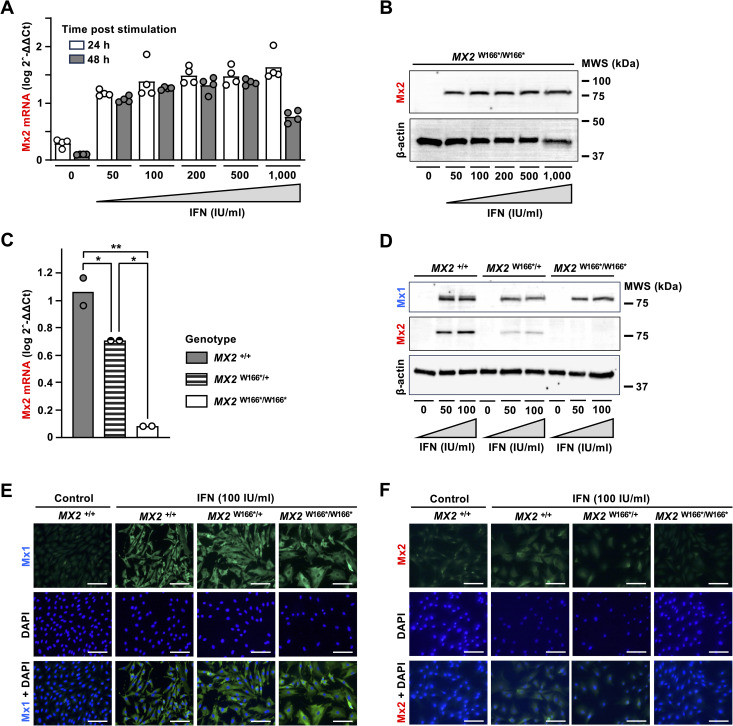
Interferon-induced expression of Mx in sheep primary fibroblasts. (A) Detection of Mx2 mRNA in wild-type (*MX2*^*+/+*^) fibroblasts 24 and 48 hours after the stimulation with various IFN-α concentrations using RT-qPCR and the 2-∆∆Ct method with *GAPDH* expression as a reference. (B) Detection of Mx2 protein in wild-type (*MX2*^*+/+*^) fibroblasts 48 h after the stimulation with various concentrations of IFN-α using Western blotting (β-actin was stained as a reference). Mx2 and β-actin proteins were labelled using a rabbit polyclonal and a mouse monoclonal antibody, respectively. (C) Interferon-induced expression of Mx2 mRNA in wild-type (*MX2*^*+/+*^) fibroblasts and fibroblasts heterozygous or homozygous for a SNP (W166*) that terminates the ORF prematurely. Cells were stimulated with 100 IU/mL IFN-α for 48 hours prior to the analysis (Mx2 mRNAs were quantified as described for panel A). Statistical significance: *, p < 0.05; **, p = 0.0027 (adjusted p-values) with Tukey’s HSD by GraphPad Prism (v. 10.5.0). (D) Interferon-induced expression of Mx1 and Mx2 proteins in IFN-stimulated wild-type fibroblasts (*MX2*^*+/+*^) and fibroblasts heterozygous or homozygous for a SNP (W166*) that terminates the ORF prematurely. Mx1 protein was labelled using a rabbit polyclonal antibody (Mx2 and β-actin were labelled as described for panel B). (E, F) Accumulation and intracellular localisation of Mx1 and Mx2 in wild-type fibroblasts (*MX2*^*+/+*^) and fibroblasts heterozygous or homozygous for a SNP (W166*) that prematurely terminates the ORF. Cells were either stimulated with 100 IU IFN-α or left untreated, fixed 48 hours after the stimulation, and immunostained using polyclonal antibodies specific for Mx1 (E) or Mx2 (F). To visualize nuclei, cellular DNA was stained using DAPI. Green, Mx-specific staining; blue, DNA staining. Scale bars, 500 µm. All experiments were conducted at least twice.

To characterise the expression of functional and non-functional *MX2* alleles, we established primary skin fibroblast cultures from wild-type sheep (*MX2* ^+/+^) and sheep that are heterozygous or homozygous for the alternative allele at W166*. At first, we conducted pilot studies using wild-type cells to determine the ability of a pan-specific human hybrid IFN-α to stimulate ovine *MX2* gene expression. Cells were grown in 6-well plates with different doses of IFN-α, and the accumulation of Mx2 mRNAs and proteins was analysed by qRT-PCR (Fig 4A) and Western blotting (Fig 4B), respectively. As anticipated, Mx2 mRNA and protein concentrations were very low in unstimulated cells, but concentrations quickly increased after the stimulation with IFN. Mx2 proteins became easily detectable after incubating cells for 48 hours with as little as 50 IU/mL IFN.

**Fig 5:**
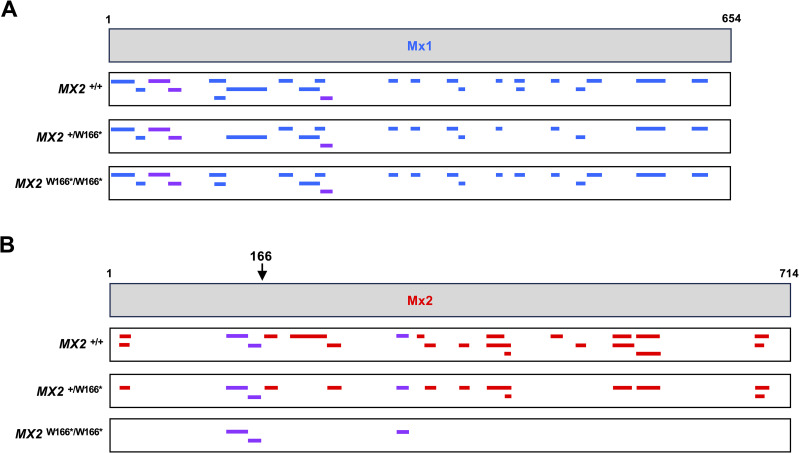
Mx protein detection by mass spectrometry. (A, B) Peptide coverage for the Mx1 protein and Mx2 protein, respectively. Wild-type fibroblasts (*MX2*^+*/*+^) and fibroblasts heterozygous or homozygous for a SNP (W166*) that terminates the ORF prematurely were stimulated with 100 IU IFN-α for 24 hours, and cell lysates were subjected to mass spectrometry. Blue, peptides with Mx1 sequences; red, peptides with Mx2 sequences; purple, peptides with sequences that exist in Mx1 and Mx2 proteins.

To characterise Mx expression in cells with defective *MX2* alleles, fibroblasts generated from wild-type sheep and sheep that were heterozygous or homozygous for the alternative allele at W166* were cultured with or without IFN for 48 hours, and whole cell lysates were analysed for the accumulation of Mx2 mRNAs (Fig 4C) and Mx2 proteins (Fig 4D). We found that the presence of the premature stop at codon 166 significantly reduced Mx2 mRNAs levels and completely abolished the expression of Mx2 proteins. We could not detect any Mx2 proteins in cells homozygous for the W166* allele, while heterozygous cells expressed approx. half as much Mx2 protein as wild-type cells. Despite a careful inspection of the Western blot membrane, we did not find bands that indicate the presence of a truncated Mx2 protein or of degradation products, an outcome that was not unexpected given that we used an Mx2-specific antibody raised against an immunogenic peptide of 50 aa with a sequence that is not found in the first 166 aa of the ovine Mx2. Furthermore, we analysed the expression of Mx1 proteins to exclude the possibility that the reduced expression of Mx2 proteins was due to defective IFN signalling and found that the presence of a defective *MX2* allele did not affect the expression of the *MX1* gene (Fig 4D and E). To corroborate our findings, we analysed the IFN-induced expression of Mx1 and Mx2 proteins using immunofluorescence, a technique that usually is more sensitive than immunoblotting. However, we could not detect Mx2-specific fluorescence in fibroblasts homozygous for the alternative allele at W166*, while cells with a functional allele stained positive for Mx2 (Fig 4E and F). Moreover, we used mass spectrometry to analyse the presence of truncated Mx2 proteins in cells with the alternative allele at W166* (as the Mx2-specific antibody that was used in our immunoblotting and immunofluorescence assays does not bind to the first 166 aa of Mx2 or to degradation products of such a truncated protein). Although we found Mx1 peptides in IFN-stimulated cells of all genotypes and Mx2 peptides in IFN-stimulated wild-type cells, we did not find any Mx2 peptides in IFN-stimulated cells with the alternative null allele at W166* (Fig 5 and S3–S5 Tables). The finding confirms the notion that the premature stop codon in the alternative allele at W166* completely abolishes protein expression, most likely through nonsense-mediated mRNA decay.

**Fig 6:**
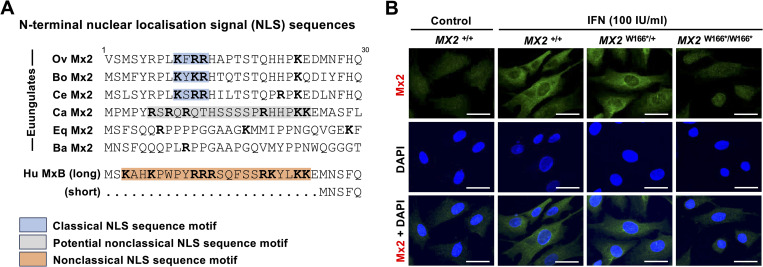
Intracellular localisation of Mx2. (A) Alignment of the N-terminal 30 amino acids of euungulate Mx2 proteins (the corresponding sequence of the human ortholog MxB is shown for comparison). Note that alternative splicing produces two isoforms of the human MxB (49). Ov, ovine (*Ovis aries*); Bo, bovine (*Bos taurus*); Ce, cervine (*Cervus elaphus*); Eq, equine (*Equus caballus*); Ba, baleen (*Balaeoptera acutorostrata*); Hu, human (*Homo sapiens*); classical NLS sequence motifs are highlighted in blue; potential and known non-classical NLS sequence motifs are highlighted in grey and orange, respectively; positively charged aa are printed in bold. (B) Sheep wild-type fibroblasts (*MX2*^*+/+*^) and fibroblasts heterozygous or homozygous for a SNP (W166*) that prematurely terminates the ORF were either stimulated with 100 IU IFN-α or left untreated, fixed 48 hours after the stimulation, and immunostained using a polyclonal antibody specific for Mx2 (green). Additionally, cells were stained with DAPI (blue). Scale bars, 25 µm.

Different Mx proteins accumulate in different cellular compartments. The ovine Mx1 protein has previously been found to accumulate in the cytoplasm [26], similar to the intracellular localisation of the human MxA protein and its non-rodent homologs. So, it was not surprising to find that the ovine Mx1 protein accumulates in the cytoplasm of IFN-stimulated fibroblasts (Fig 4E). The intracellular location of the Mx2 protein is less clear. The human homolog of the ovine Mx2 protein, MxB, has a ‘non-conventional’ NLS in the first 25 aa of the protein, which directs the protein to the nucleus [47] and, together with the next 65 aa of the N-terminal domain, is critical for the interaction with nuclear pore proteins and viral nucleocapsids [30,48–51]. When we analysed the sequence of the ovine Mx2 protein, we identified a ‘conventional’ NLS near the N-terminus and found that this NLS is conserved among a monophyletic group of ungulates that include bovids (sheep, cattle, antelopes and others) and cervids (true deer) but not camels, horses and whales (Fig 6A). When Busnadiego and co-workers [34] analysed the cellular localisation of a recombinant HA-tagged ovine Mx2, the protein accumulated in the cytoplasm with no apparent interaction with the nuclear membrane or nuclear pores. Our observations in IFN-treated fibroblasts are somewhat different. Although we found that the protein mostly accumulates in the cytoplasm, some cells showed a weak perinuclear staining (Fig 6B). The reason for the observed difference in the intracellular localisation of ovine Mx2 is not clear, and further studies are required to determine how much of the protein is transported to the nuclear membrane and if a nuclear and/or perinuclear localisation is required for antiviral activity.

## Discussion

### Domestication can lead to a loss of functional Mx alleles

Mx proteins play a key role in the innate immune defence against virus infections. All vertebrate species that have been analysed so far possess functional Mx genes with the notable exception of toothed whales [52]. This, however, does not mean that all individuals of a species possess functional genes, and it seems that domestication can significantly increase the presence of non-functional alleles. For example, almost all strains of laboratory mice (*Mus musculus domesticus*) are Mx1-negative [6], although most wild mice (*M. musculus* and related species such as *Mus spretus*) are Mx1-positive [53–55]. The reason for the wide-spread loss of functional Mx genes in laboratory mice is not known but likely is a consequence of domestication and inbreeding. We think that the phenomenon might be common and decided to analyse the integrity of ovine Mx genes.

Sheep were domesticated approx. 10,000 years ago and numerous breeds have since spread far beyond the geographic origin of the species [56]. Small population sizes during the domestication process and during colonisation of new areas along with an ongoing artificial selection for breed-defining traits provided ample opportunity for an unintentional accumulation of deleterious variants [57]. When we analysed sequence data from 68 sheep, representing 43 breeds from 19 countries, we found two non-functional *MX2* alleles with premature stop codons (either W166* or Q329Sfs7*). The data suggest that non-functional alleles exist in a wide range of breeds including Santa Inês, Swiss White Alpine, Finnsheep, Cheviot, Gulf Coast Native, Valais Blacknose, Texel, Ethiopian Menz, Awassi and Sakiz. Most of the breeds in our study are represented by only one or two sheep, which means that non-functional alleles could be more widespread. A subsequent genotyping of Australian sheep showed that this is indeed the case. We found the W166* allele in two Merino flocks although none of the four Merinos that were sampled by the International Genomics Consortium carried the defective allele. A more comprehensive analysis of Australian Merinos is currently underway. The results of this and similar studies in other breeds will inform the industry about the allele frequency of non-functional Mx genes. If such alleles are indeed widespread in production animals, breeders may consider improving the innate resistance against virus infections by eliminating non-functional Mx genes.

### SNPs that impact the Mx protein-mediated antiviral defence

In this paper, we focus on the characterisation of two SNPs that completely abolish the expression of Mx2 proteins. We found additional SNPs in the ORF of *MX1* and *MX2* (Table 1), but we currently do not know whether they affect the antiviral activity of the encoded proteins. Mx proteins oligomerise, and nonfunctional or abnormally structured Mx proteins that interfere with oligomerisation are often dominant-negative, i.e., the expression of a nonfunctional Mx protein can disrupt the antiviral function of a wild-type protein, even if the wild-type protein is abundantly expressed [58]. Recent work by Chen and co-workers identified a strong association between zoonotic avian influenza virus infections and rare SNPs in the human *MX1* gene [59]. Nearly all of the antivirally inactive protein variants that were identified in that study exerted a dominant-negative effect, suggesting a null phenotype in heterozygous carriers. Thus, SNPs that inactivate the antiviral activity of an Mx protein but not its expression can be even more devastating than those that abolish expression. It will be interesting to determine whether any of the SNPs that we identified affect the activity of Mx proteins and act in a dominant-negative manner. But before this can be achieved, more research is required to determine which viruses are inhibited by ovine Mx1 and Mx2 proteins and to establish functional assays to test the activities of ovine Mx variants. One might speculate that the ovine Mx2 protein inhibits sheep pathogens such as the *Ovine gammaherpesvirus-2* or the *Jaagsiekte sheep retrovirus* given that the ortholog protein in humans inhibits the replication of herpes- and retroviruses [29,32,33].

### A loss of functional Mx alleles may affect pregnancy testing in ruminants

The discovery of SNPs that completely abolish expression of Mx proteins has implications for using Mx expression as a reproductive biomarker [16,19]. The presence of the SNPs W166* and Q329Sfs7* in the Mx2 coding sequences of 13 breeds from several countries suggests that the inability to express Mx2 proteins is widely spread. Although a loss of Mx2 expression has little if any effect on the fertility (as observed in this study), an ewe that is homozygous for W166* will not test positive in a pregnancy test that is based on Mx2 expression. In fact, Mauffré and co-workers found that quantifying the expression of Mx proteins (and that of other interferon-stimulated genes) in peripheral blood monocytes is not a reliable marker for the detection of early pregnancies in sheep [19]. There is more than one explanation for this phenomenon, but it is tempting to speculate that some of the animals carried non-functional Mx alleles. Similarly, homozygosity for a null allele that abolishes expression would affect the value of Mx proteins as markers for virus replication. For example, Mx proteins expression has been used in the differential diagnosis of viral and bacterial diseases in children ahead of a treatment with antibiotics [60–62]. Adopting this approach for diagnosing viral diseases in sheep would be difficult in flocks that carry alleles with W166* or similar SNPs.

### Functional Mx alleles in livestock improve animal health and may prevent the spread of zoonotic viruses

Mx proteins significantly contribute to the innate resistance against viral infections, and non-functional genes do not only affect the health of the carrier but also affect the population health of sheep and even other species. The detection of highly pathogenic avian influenza viruses in dairy cattle [63] and, most recently also in sheep (https://www.gov.uk/government/news/influenza-of-avian-origin-confirmed-in-a-sheep-in-yorkshire) is a case in point. Animals with non-functional *MX1* alleles are more vulnerable to influenza infections, which not only affects animal health and productivity but also increases the risk of zoonotic infections. Infected animals produce more viruses, which increases the risks of cross-species transmissions. It is therefore recommended that the breeding of livestock seeks to minimise the risk of losing of functional alleles that encode key mediators of the innate immune response against viruses.

## Conclusions

The breeding of sheep and other farmed animals is part of the ‘One Health’ approach and thus comes with great responsibility for both animal and human health because of livestock-associated zoonotic diseases. A screening of sheep genome sequences revealed the existence of two single nucleotide polymorphisms in the *MX2* gene that affect protein expression and functionality. A subsequent genotyping of Australian sheep confirmed that the presence of such alleles in production animals. Existing breeding programs for sheep and other livestock should be adjusted to reduce the frequency of non-functional Mx alleles.

## Materials and methods

### Animal ethics

All experiments involving animals were carried out in strict accordance with the recommendations in the Australian code of practice for the care and use of animals for scientific purposes of the National Health and Medical Research Council of Australia. Experiments were approved and performed under guidelines set out by the F.D. McMaster Animal Ethics Committee, CSIRO Agriculture and Food, under the oversight of the NSW Department of Primary Industries (ARA 15/11, 17/11 and 18/03).

### Sheep

Blood samples for DNA analysis were obtained from 141 animals derived from two selection lines. These animals are maintained with paternal parentage records at the CSIRO research station Chiswick, which is part of the CSIRO F.D. McMaster Laboratory in Armidale (NSW, Australia). The two lines are referred to as the *Haemonchus* selection flock (HSF) [44] and the *Trichostrongylus* selection flock (TSF) [45]. HSF and TSF were established from fine wool Merino and medium wool Peppin Merinos, respectively (for a description of the founder ‘strains’, see [46]). More than 30 years of selection for resistance and susceptibility to gastrointestinal parasites has produced highly resistant (HSFR and TSFR) and highly susceptible (HSFS and TSFS) lines, respectively; these lines have been studied intensively [64]. All animals were given numbers (e.g., 2012A0020); information on the naming convention for individual animals can be accessed online [65].

To study the effect of non-functional *MX2* alleles on fertility and early development, we set up single-sire-mated groups of ewes, genotyped the sires, dams and lambs, assessed pregnancy by ultrasound examination 80 days after mating, identified and weighed the lambs at birth and followed their growth rate for approx. 30 days. These lambs were also inspected visually to detect any obvious differences between wild-type and Mx2-negative animals.

The frequencies of alleles and genotypes between groups were statistically compared using chi-squared tests (α = 0.5) and compared to Hardy-Weinberg expectations using chi-squared tests (α = 0.5). Chi-squared tests were also used to compare the proportions of ewes of different genotypes that had live births or became pregnant and the proportions of male and female offspring for rams of different genotypes. Analysis of variance was used to determine statistical significance of the lamb weights and growth rates relative to ram genotype, dam genotype and lamb genotype. All statistical analysis was conducted using Genstat (24^th^ edition), lamb weights and growth rates were normally distributed, and no multiple testing corrections were used for these small datasets (α = 0.05).

### Blood sampling and DNA preparation

Animals were subjected to blood sampling by jugular venepuncture (10-mL samples, collected and supplemented with 1.8 mg/mL K_2_EDTA using BD Vacutainers (Becton Dickinson, Franklin Lakes, NJ). Blood was centrifuged at 3,000 g for 20 min at 4°C; buffy coat cells were collected using a disposable Pasteur pipette and stored at −20˚C before subsequent DNA preparation. Genomic DNA was extracted from 100-µl subsamples of frozen buffy coat cells using the GenElute Blood Genomic DNA kit (Sigma-Aldrich, St. Louis, MO) according to the manufacturer’s instructions.

### Cells

Primary fibroblasts cultures were established using skin samples of adult sheep with or without functional *MX2* alleles. Prior to the sampling, wool surrounding the biopsy area was gently removed using a handheld electric shaver, and the area was cleaned using Chlorhex C (50 mg/mL chlorhexidine gluconate; Jurox, Rutherford, NSW, Australia) before 1 mL of 2% Lignocaine (Sigma-Aldrich, St. Louis, MO, USA), a local anaesthetic, was intradermally injected to induce the formation of a controlled blister (bleb) on the skin surface. A biopsy punch with a diameter of 0.8 cm was taken from skin directly over the bleb, subcutaneous tissue was transferred into a 15-mL tube containing 2 mL of Hanks’ balanced salt solution (HBSS) supplemented with antibiotic-antimycotic agents (Anti-Anti; Thermo Fisher Scientific, USA), washed twice with HBSS, and finely minced using a pair of sterile scalpels. The resulting pieces were carefully transferred into 2-mL tubes preloaded with 1 mL of a collagenase (1,000 U/mL; Sigma-Aldrich) in HBSS. The tubes were then placed on a tube rotator and incubated at 37 °C for 3 hours. The digested tissues samples were passed through a sterile 70-μm cell strainer, transferred to 2-mL tubes with 0.5 mL of HBSS and centrifuged at 2,000 × g for 5 min. The pelleted cells were washed again, re-suspended in 0.5 mL of DMEM (Dulbecco’s Modified Eagle Medium) (Thermo Scientific), supplemented with non-essential amino acids, penicillin, streptomycin (Sigma-Aldrich) and 10% foetal calf serum (FCS) (GIBCO, Waltham, MA, USA), seeded into a single well of a 6-well plate pre-coated with collagen and incubated with 5% CO_2_ at 37 °C. After 3 days, the medium was replaced, and the plate was incubated for an additional 3 days, during which the cells achieved 80–90% confluency. The cells were subsequently propagated using standard cell culture protocols.

### Cytokines

The Universal Type I IFN (PBL Assay Science, Piscataway, NJ, USA), a human IFN-α hybrid protein with strong pan-species activities, was used to induce Mx protein expression in sheep fibroblasts. Cells were incubated with IFN for either 24 or 48 hours without refreshing the media.

### Data mining using whole genome sequences

Whole genome sequence data of 68 sheep representing 43 breeds from 19 countries was generated by the International Sheep Genomics Consortium, a partnership of researchers and funding agencies (http://www.sheephapmap.org/) [36,43]. The full genome data has been deposited in a public repository (unfiltered, https://doi.org/10.25919/5d3a234da46eb; filtered https://doi.org/10.25919/5d39e494936c6). The genome coordinates for *MX1* and *MX2* are chromosome 1: 259,735,321–259,763,804 and chromosome 1: 259,686,071–259,717,801, respectively (based on Oar_v3.1:CM001582.1).

The SAMtools suite (http://samtools.sourceforge.net) [66] was used to extract reads aligned to genome regions (*O. aries* genome sequence NC_019458.2) that correspond to ovine *MX1* (Gene ID: 443146) and *MX2* (Gene ID: 780441) sequences. The reads obtained were re-aligned to *MX1* or *MX2* sequences using Geneious v. 10.1.3 (Biomatters, Auckland, New Zealand [67] and variant calling was undertaken. Potential variants were further analysed to produce lists of variant sequences that fulfil the following criteria: (*i*) the nucleotide variation was observed in regions of 10-fold or higher read coverage in each individual dataset; (*ii*) the observed nucleotide variation with a minor allele read frequency was greater than 0.45 for at least one individual; (*iii*) homozygotes all had one of the two possible nucleotides (or gaps) observed in the heterozygotes, or were all homozygous for the non-reference allele; and (*iv*) the observed nucleotide variation was of the same type in at least two heterozygous individuals and was the same type in all heterozygous individuals considered. To ensure that the frequency of variants was not overly affected by sample size, a “suitable coverage region” criterion was added. Suitable coverage regions were defined as the part of the alignment where the total number of reads across all individuals with a minimum read coverage of 10 exceeded 99 reads. All variants reported in this manuscript met the criteria outlined above and raw SNP predictions can be accessed online at https://doi.org/10.25919/6bgq-8d50. Variant positions are given relative to the inferred open reading frame obtained by comparing the cDNA sequence for *MX1* and *MX2* (GenBank accession no. JN377734 and AY859475, respectively) with the *O. aries* genome sequence (accession no. NC_056054). Of note, the published cDNA sequence for *MX2* (GenBank accession no. AY859475) varies from the predicted coding sequence in the genomic sequence (the genomic sequence suggests two extra codons at the start of the ORF). In this paper, positions of *MX1* and *MX2* sequences variants are given relative to the predicted coding sequence of the relevant genomic and cDNA sequences.

### Genotyping

A region encompassing exon 5 of the ovine *MX2* gene was amplified by PCR using primer F1 (5’-GGTGCAGTCAGAAGGGAAAA-3’) together with either reverse primer R2 (5’-AAGGAGCCGACTTGCAGAT-3’) or reverse primer R3 (5’-CTGAGTGACCGCTGATGACC-3’). Amplicons were purified using the QIAquick PCR purification kit (Qiagen, Hilden, Germany) according to the manufacturer’s instructions. For forward direction dye terminator sequencing, we used F1-R2 amplicons and the F1b primer (5’-TCAGAAGGGAAAAGAAAAGGG-3’), and for reverse sequencing, we used F1-R3 amplicons and the R3 primer. Cycle sequencing was conducted using the GenomeLab DTCS quick start kit (Beckman Coulter, Brea, CA) and a CEQ 8000 capillary electrophoresis system (Beckman Coulter) according to the manufacturer’s instructions with the following thermocycler settings: 95 °C for 15 min, followed by 30 cycles at 95 °C for 40 s, 60 °C for 40 s and at 72 °C for 60 s. Sequencing traces were subjected to quality control with the manufacturer’s sequencing software and interrogated manually, and where required, reactions were repeated. Sequences that passed these tests were analysed for the presence of overlying peaks in the trace and edited where required. Forward and reverse sequences were compared, and in cases of a disagreement, traces were re-analysed; if the re-analysis did not resolve the discrepancy, a new PCR was performed, and sequencing was repeated. Comparison of genotypes and allele frequencies, and adherence to Hardy-Weinberg expectations were undertaken using chi-square analysis in Microsoft Excel. ANOVA was used to compare live weights and growth between groups of lambs by genotype and by sex using Genstat (24^th^ edition, VSN International, Hemel Hempstead, Hertfordshire, UK).

### Rapid amplification of cDNA ends (RACE)

To determine the 5′ UTR sequence of the *MX2* gene, a 5′ RACE was conducted using the 5’ RACE System for Rapid Amplification of cDNA Ends, version 2.0 (Thermo Fisher Scientific) according to manufacturer’s instructions. Wild-type fibroblasts were cultured with 100 U/mL IFN for 2 days, and RNA was isolated with RNeasy Plus Kit for RNA Isolation (Qiagen). An Mx2-specific primer (5’-GTTGTTTTCGGGCCCCTTTG-3’) and the SuperScript II Reverse Transcriptase (Thermo Fisher Scientific) was used to synthesise first-strand cDNA according to manufacturer’s instructions before a homopolymeric tail was added to the 3’-end of the cDNA using TdT and dCTP. Next, a PCR was carried out using the abridged anchor primer (5’-GGCACGCGTCGACTAGTACGGGIIGGGIIGGGIIG-3’) of the 5’ RACE kit with a Mx2-specific primer (5’-AGTTGTTGGGAAGGAAGTCCG-3’) and the following thermocycler settings: 94 °C for 180 s, followed by 35 cycles at 94 °C for 10 s, 55 °C for 30 s and 72 °C for 60 s. The PCR product was further amplified using the universal amplification primer of the 5’ RACE kit, another Mx2-specific primer (5’-ACTCATTGTCTGCCCCAGTG-3’) and the following thermocycler settings: 94 °C for 180 s, followed by 35 cycles at 94 °C for 10 s, 55 °C for 30 s and 72 °C for 60 s. The final PCR products were then purified and submitted to Australian Genome Research Facility (AGRF) for Sanger sequencing.

### Quantitative PCR

RNA was isolated from fibroblasts with or without the premature stop codon W166* that had been cultured with 100 IU/mL IFN-α for 24 or 48 hours using the RNeasy Plus Kit for RNA isolation (Qiagen, Hilden, Germany), and cDNA was synthesised using Invitrogen SuperScript IV (Thermo Fisher Scientific) according to manufacturer’s instructions. A qRT PCR was carried out using a PowerTrack SYBR Green Master Mix for qPCR (Thermo Fisher Scientific) with Mx2-specific primers (5’-AAGTACGAGGAGAAGGTGCG-3’ (forward), 5’-GTTACGTTGCGGTAGGTGAT-3’ (reverse)) or GAPDH-specific primers (5’-TCGGAGTGAACGGATTTGGC-3’(forward), 5’-TTCCCGTTCTCAGCC TTGAC-3’(reverse)) on the CFX96 Touch Real-Time PCR Detection System (Bio-Rad, Hercules, CA, USA) with the following thermocycler settings: 94 °C for 180 s, followed by 35 cycles at 94 °C for 10 s, 55 °C for 30 s and at 72 °C for 60 s. Two independent qRT PCRs with four technical replicates were performed for each gene. The relative expression of the *MX2* gene was calculated against the expression of the house-keeping gene *GAPDH* according to 2^-∆∆ct^ method.

### Immunoblotting

Whole cell lysates were prepared in RIPA buffer (Thermo Fisher Scientific) with protease inhibitor (Roche, Basel, Switzerland), and samples containing equal amounts of proteins were subjected to sodium dodecyl sulfate-polyacrylamide gel electrophoresis and blotted to PVDF membranes [68]. Blots were labelled using Mx1- and Mx2-specific rabbit polyclonal antibodies (Cat# PA5–22101 and Cat# PA5–102005, respectively; obtained from Thermo Fisher Scientific); the β-actin-specific rabbit mAb D6A8 (Cat# 8457) was obtained from Cell Signaling Technology (Danvers, MA, USA) and the HRP-conjugated sheep anti-rabbit polyclonal antibody (Cat# ab6795, RRID:AB_955446) was obtained from Abcam (Waltham, MA, USA). Secondary antibody-specific signals were generated using ECL solutions (Bio-Rad) and imaged using the Vü system (Pop-Bio Imaging, Cambridge, UK).

### Immunofluorescence

Fibroblasts grown on a glass coverslip in the presence or absence of type I IFN for 48 hours were fixed and permeabilised with 100% chilled methanol. The coverslip was then blocked with 5% goat serum and incubated overnight at 4 °C with either Mx1- or Mx2-specific primary antibodies (described in the paragraph above) followed by an incubation with an Alexa Fluor 488-conjugated, rabbit-specific goat antibody (Abcam, Cat# ab150077). The coverslip was mounted with Fluoroshield containing DAPI (Abcam, Cat# ab104139). Fluorescence was analysed using an Eclipse T*i* microscope and the NIS-Elements D4 software (Nikon, Tokyo, Japan) or a Leica SP8 Lightning confocal microscope and the LAS AF software (Leica Microsystems, Wetzlar, Germany).

### Mass spectrometry

#### Protein extraction and quantification.

Protein samples were prepared using a previously method with modifications [69]. After precipitation with acetone, proteins were reduced, alkylated and digested with trypsin. The trypsin digested samples were analysed using previously published methods [69–71]. Each sample (500 ng) was desalted and separated using a nano LC system (Ultimate^TM^ 3000, Thermo Fisher Scientific). Mass spectra (MS1) and tandem mass spectra (MS/MS) were collected by an Orbitrap Fusion mass spectrometer (Thermo Fisher Scientific).

#### Analysis.

Publicly available MaxQuantGui [72] was used to identify peptides/proteins and quantify the relative abundance of proteins. The spectrum data was analysed against the UniProt *O. aries* database (Proteome ID: UP000002356; 23,108 sequences). Relative abundance was calculated from precursor abundance intensity and normalised to the total peptide amount.

## Supporting information

S1 Table Impact predictions of amino acid substitutions in ovine Mx protein variants. (PDF)

S2 Table Birth weight and weight gain of lambs with functional and nonfunctional *MX2* alleles. (PDF)

S3 Table Identification of Mx1 peptides by LC-MS. (PDF)

S4 Table Identification of Mx2 peptides by LC-MS. (PDF)

S5 Table Identification of Mx1/Mx2 peptides by LC-MS. (PDF)

S1 Fig Regulatory elements in ovine Mx gene promoters. (PDF)

S1 File Suppl Material S1 (raw images) (17-10-25). (PDF)

## References

[1] Langley CA, Dietzen PA, Emerman M, Tenthorey JL, Malik HS. Antiviral Mx proteins have an ancient origin and widespread distribution among eukaryotes. Proc Natl Acad Sci U S A. 2025;122(4):e2416811122. doi: 10.1073/pnas.2416811122 39854241 PMC11789081

[2] Haller O, Staeheli P, Schwemmle M, Kochs G. Mx GTPases: dynamin-like antiviral machines of innate immunity. Trends Microbiol. 2015;23(3):154–63. doi: 10.1016/j.tim.2014.12.003 25572883

[3] Wu X, Dao Thi VL, Huang Y, Billerbeck E, Saha D, Hoffmann H-H, et al. Intrinsic Immunity Shapes Viral Resistance of Stem Cells. Cell. 2018;172(3):423-438.e25. doi: 10.1016/j.cell.2017.11.018 29249360 PMC5786493

[4] Verhelst J, Hulpiau P, Saelens X. Mx proteins: antiviral gatekeepers that restrain the uninvited. Microbiol Mol Biol Rev. 2013;77(4):551–66. doi: 10.1128/MMBR.00024-13 24296571 PMC3973384

[5] Betancor G. You Shall Not Pass: MX2 Proteins Are Versatile Viral Inhibitors. Vaccines (Basel). 2023;11(5):930. doi: 10.3390/vaccines11050930 37243034 PMC10224399

[6] Staeheli P, Grob R, Meier E, Sutcliffe JG, Haller O. Influenza virus-susceptible mice carry Mx genes with a large deletion or a nonsense mutation. Mol Cell Biol. 1988;8(10):4518–23. doi: 10.1128/mcb.8.10.4518-4523.1988 2903437 PMC365527

[7] Horisberger MA, Staeheli P, Haller O. Interferon induces a unique protein in mouse cells bearing a gene for resistance to influenza virus. Proc Natl Acad Sci U S A. 1983;80(7):1910–4. doi: 10.1073/pnas.80.7.1910 6188159 PMC393720

[8] Arnheiter H, Skuntz S, Noteborn M, Chang S, Meier E. Transgenic mice with intracellular immunity to influenza virus. Cell. 1990;62(1):51–61. doi: 10.1016/0092-8674(90)90239-b 2194673

[9] Kolb E, Laine E, Strehler D, Staeheli P. Resistance to influenza virus infection of Mx transgenic mice expressing Mx protein under the control of two constitutive promoters. J Virol. 1992;66(3):1709–16. doi: 10.1128/JVI.66.3.1709-1716.1992 1371172 PMC240917

[10] Thimme R, Frese M, Kochs G, Haller O. Mx1 but not MxA confers resistance against tick-borne Dhori virus in mice. Virology. 1995;211(1):296–301. doi: 10.1006/viro.1995.1404 7645224

[11] Haller O, Frese M, Rost D, Nuttall PA, Kochs G. Tick-borne thogoto virus infection in mice is inhibited by the orthomyxovirus resistance gene product Mx1. J Virol. 1995;69(4):2596–601. doi: 10.1128/JVI.69.4.2596-2601.1995 7884909 PMC188937

[12] Jiang Y, Xie M, Chen W, Talbot R, Maddox JF, Faraut T, et al. The sheep genome illuminates biology of the rumen and lipid metabolism. Science. 2014;344(6188):1168–73. doi: 10.1126/science.1252806 24904168 PMC4157056

[13] Charleston B, Stewart HJ. An interferon-induced Mx protein: cDNA sequence and high-level expression in the endometrium of pregnant sheep. Gene. 1993;137(2):327–31. doi: 10.1016/0378-1119(93)90029-3 7507876

[14] Ott TL, Yin J, Wiley AA, Kim HT, Gerami-Naini B, Spencer TE, et al. Effects of the estrous cycle and early pregnancy on uterine expression of Mx protein in sheep (Ovis aries). Biol Reprod. 1998;59(4):784–94. doi: 10.1095/biolreprod59.4.784 9746726

[15] Spencer TE, Stagg AG, Ott TL, Johnson GA, Ramsey WS, Bazer FW. Differential effects of intrauterine and subcutaneous administration of recombinant ovine interferon tau on the endometrium of cyclic ewes. Biol Reprod. 1999;61(2):464–70. doi: 10.1095/biolreprod61.2.464 10411528

[16] Yankey SJ, Hicks BA, Carnahan KG, Assiri AM, Sinor SJ, Kodali K, et al. Expression of the antiviral protein Mx in peripheral blood mononuclear cells of pregnant and bred, non-pregnant ewes. J Endocrinol. 2001;170(2):R7-11. doi: 10.1677/joe.0.170r007 11479146

[17] Assiri AM, Ott TL. Cloning and characterizing of the ovine MX1 gene promoter/enhancer region. Dev Comp Immunol. 2007;31(8):847–57. doi: 10.1016/j.dci.2006.12.004 17275905

[18] Johnson GA, Joyce MM, Yankey SJ, Hansen TR, Ott TL. The Interferon Stimulated Genes (ISG) 17 and Mx have different temporal and spatial expression in the ovine uterus suggesting more complex regulation of the Mx gene. J Endocrinol. 2002;174(2):R7–11. doi: 10.1677/joe.0.174r007 12176677

[19] Mauffré V, Grimard B, Eozenou C, Inghels S, Silva L, Giraud-Delville C, et al. Interferon stimulated genes as peripheral diagnostic markers of early pregnancy in sheep: a critical assessment. Animal. 2016;10(11):1856–63. doi: 10.1017/S175173111600077X 27150201

[20] Racicot K, Ott T. The myxovirus resistance protein, MX1, interacts with tubulin beta in uterine glandular epithelial cells. Am J Reprod Immunol. 2011;65(1):44–53. doi: 10.1111/j.1600-0897.2010.00885.x 20584010

[21] Toyokawa K, Carling SJ, Ott TL. Cellular localization and function of the antiviral protein, ovine Mx1 (oMx1): I. Ovine Mx1 is secreted by endometrial epithelial cells via an “unconventional” secretory pathway. Am J Reprod Immunol. 2007;57(1):13–22. doi: 10.1111/j.1600-0897.2006.00444.x 17156187

[22] Toyokawa K, Leite F, Ott TL. Cellular localization and function of the antiviral protein, ovine Mx1 (oMx1): II. The oMx1 protein is a regulator of secretion in an ovine glandular epithelial cell line. Am J Reprod Immunol. 2007;57(1):23–33. doi: 10.1111/j.1600-0897.2006.00439.x 17156188

[23] Ealy AD, Wooldridge LK. The evolution of interferon-tau. Reproduction. 2017;154(5):F1–10. doi: 10.1530/REP-17-0292 28982935

[24] Hansen TR, Sinedino LDP, Spencer TE. Paracrine and endocrine actions of interferon tau (IFNT). Reproduction. 2017;154(5):F45–59. doi: 10.1530/REP-17-0315 28982937

[25] Casano AB, Menchetti L, Trabalza-Marinucci M, Riva F, De Matteis G, Brecchia G, et al. Gene expression of pregnancy-associated glycoproteins-1 (PAG-1), interferon-tau (IFNt) and interferon stimulated genes (ISGs) as diagnostic and prognostic markers of maternal-fetal cellular interaction in buffalo cows. Theriogenology. 2023;209:89–97. doi: 10.1016/j.theriogenology.2023.06.028 37379587

[26] Racicot K, Schmitt A, Ott T. The myxovirus-resistance protein, MX1, is a component of exosomes secreted by uterine epithelial cells. Am J Reprod Immunol. 2012;67(6):498–505. doi: 10.1111/j.1600-0897.2012.01109.x 22574859

[27] Frese M, Kochs G, Meier-Dieter U, Siebler J, Haller O. Human MxA protein inhibits tick-borne Thogoto virus but not Dhori virus. J Virol. 1995;69(6):3904–9. doi: 10.1128/JVI.69.6.3904-3909.1995 7745744 PMC189115

[28] Haller O, Frese M, Kochs G. Mx proteins: mediators of innate resistance to RNA viruses. Rev Sci Tech. 1998;17(1):220–30. doi: 10.20506/rst.17.1.1084 9638812

[29] Schoggins JW, Wilson SJ, Panis M, Murphy MY, Jones CT, Bieniasz P, et al. A diverse range of gene products are effectors of the type I interferon antiviral response. Nature. 2011;472(7344):481–5. doi: 10.1038/nature09907 21478870 PMC3409588

[30] Wang Y-X, Niklasch M, Liu T, Wang Y, Shi B, Yuan W, et al. Interferon-inducible MX2 is a host restriction factor of hepatitis B virus replication. J Hepatol. 2020;72(5):865–76. doi: 10.1016/j.jhep.2019.12.009 31863794

[31] Yi D-R, An N, Liu Z-L, Xu F-W, Raniga K, Li Q-J, et al. Human MxB Inhibits the Replication of Hepatitis C Virus. J Virol. 2018;93(1):e01285-18. doi: 10.1128/JVI.01285-18 30333168 PMC6288330

[32] Crameri M, Bauer M, Caduff N, Walker R, Steiner F, Franzoso FD, et al. MxB is an interferon-induced restriction factor of human herpesviruses. Nat Commun. 2018;9(1):1980. doi: 10.1038/s41467-018-04379-2 29773792 PMC5958057

[33] Schilling M, Bulli L, Weigang S, Graf L, Naumann S, Patzina C, et al. Human MxB Protein Is a Pan-herpesvirus Restriction Factor. J Virol. 2018;92(17):e01056-18. doi: 10.1128/JVI.01056-18 29950411 PMC6096802

[34] Busnadiego I, Kane M, Rihn SJ, Preugschas HF, Hughes J, Blanco-Melo D, et al. Host and viral determinants of Mx2 antiretroviral activity. J Virol. 2014;88(14):7738–52. doi: 10.1128/JVI.00214-14 24760893 PMC4097781

[35] Larruskain A, Esparza-Baquer A, Minguijón E, Juste RA, Jugo BM. SNPs in candidate genes MX dynamin-like GTPase and chemokine (C-C motif) receptor-5 are associated with ovine pulmonary adenocarcinoma progression in Latxa sheep. Anim Genet. 2015;46(6):666–75. doi: 10.1111/age.12351 26365162

[36] Kijas JW, Townley D, Dalrymple BP, Heaton MP, Maddox JF, McGrath A, et al. A genome wide survey of SNP variation reveals the genetic structure of sheep breeds. PLoS One. 2009;4(3):e4668. doi: 10.1371/journal.pone.0004668 19270757 PMC2652362

[37] Gérardin JA, Baise EA, Pire GA, Leroy MP-P, Desmecht DJ-M. Genomic structure, organisation, and promoter analysis of the bovine (Bos taurus) Mx1 gene. Gene. 2004;326:67–75. doi: 10.1016/j.gene.2003.10.006 14729264

[38] Babiker HAE, Saito T, Nakatsu Y, Takasuga S, Morita M, Sugimoto Y, et al. Molecular cloning, polymorphism, and functional activity of the bovine and water buffalo Mx2 gene promoter region. Springerplus. 2016;5(1):2109. doi: 10.1186/s40064-016-3729-5 28066698 PMC5179478

[39] Yamada K, Nakatsu Y, Onogi A, Takasuga A, Sugimoto Y, Ueda J, et al. Structural and functional analysis of the bovine Mx1 promoter. J Interferon Cytokine Res. 2009;29(4):217–26. doi: 10.1089/jir.2008.0069 19203250

[40] Hug H, Costas M, Staeheli P, Aebi M, Weissmann C. Organization of the murine Mx gene and characterization of its interferon- and virus-inducible promoter. Mol Cell Biol. 1988;8(8):3065–79. doi: 10.1128/mcb.8.8.3065-3079.1988 2974922 PMC363533

[41] Chang KC, Hansen E, Foroni L, Lida J, Goldspink G. Molecular and functional analysis of the virus- and interferon-inducible human MxA promoter. Arch Virol. 1991;117(1–2):1–15. doi: 10.1007/BF01310488 1706589

[42] Thomas AV, Palm M, Broers AD, Zezafoun H, Desmecht DJ-M. Genomic structure, promoter analysis, and expression of the porcine (Sus scrofa) Mx1 gene. Immunogenetics. 2006;58(5–6):383–9. doi: 10.1007/s00251-006-0109-2 16738935

[43] Naval-Sanchez M, Nguyen Q, McWilliam S, Porto-Neto LR, Tellam R, Vuocolo T, et al. Sheep genome functional annotation reveals proximal regulatory elements contributed to the evolution of modern breeds. Nat Commun. 2018;9(1):859. doi: 10.1038/s41467-017-02809-1 29491421 PMC5830443

[44] Woolaston RR, Piper LR. Selection of Merino sheep for resistance to Haemonchus contortus: genetic variation. Anim Sci. 1996;62(3):451–60. doi: 10.1017/s1357729800014995

[45] Windon RG. Genetic control of resistance to helminths in sheep. Vet Immunol Immunopathol. 1996;54(1–4):245–54. doi: 10.1016/s0165-2427(96)05710-8 8988871

[46] Dolling CHS. Sheep and wool production in the different ecological regions in Australia with emphasis on developments in breeding. S Afr J Anim Sci. 1975;5:181–7.

[47] Melén K, Keskinen P, Ronni T, Sareneva T, Lounatmaa K, Julkunen I. Human MxB protein, an interferon-alpha-inducible GTPase, contains a nuclear targeting signal and is localized in the heterochromatin region beneath the nuclear envelope. J Biol Chem. 1996;271(38):23478–86. doi: 10.1074/jbc.271.38.23478 8798556

[48] Goujon C, Greenbury RA, Papaioannou S, Doyle T, Malim MH. A triple-arginine motif in the amino-terminal domain and oligomerization are required for HIV-1 inhibition by human MX2. J Virol. 2015;89(8):4676–80. doi: 10.1128/JVI.00169-15 25673704 PMC4442396

[49] Goujon C, Moncorgé O, Bauby H, Doyle T, Barclay WS, Malim MH. Transfer of the amino-terminal nuclear envelope targeting domain of human MX2 converts MX1 into an HIV-1 resistance factor. J Virol. 2014;88(16):9017–26. doi: 10.1128/JVI.01269-14 24899177 PMC4136259

[50] Mitchell PS, Young JM, Emerman M, Malik HS. Evolutionary analyses suggest a function of MxB immunity proteins beyond lentivirus restriction. PLoS Pathog. 2015;11(12):e1005304. doi: 10.1371/journal.ppat.1005304 26658285 PMC4687636

[51] Moschonas GD, Delhaye L, Cooreman R, Hüsers F, Bhat A, Stylianidou Z, et al. MX2 forms nucleoporin-comprising cytoplasmic biomolecular condensates that lure viral capsids. Cell Host Microbe. 2024;32(10):1705–24.e14. doi: 10.1016/j.chom.2024.09.002 39389033

[52] Braun BA, Marcovitz A, Camp JG, Jia R, Bejerano G. Mx1 and Mx2 key antiviral proteins are surprisingly lost in toothed whales. Proc Natl Acad Sci U S A. 2015;112(26):8036–40. doi: 10.1073/pnas.1501844112 26080416 PMC4491785

[53] Haller O, Acklin M, Staeheli P. Influenza virus resistance of wild mice: wild-type and mutant Mx alleles occur at comparable frequencies. J Interferon Res. 1987;7(5):647–56. doi: 10.1089/jir.1987.7.647 3681017

[54] Jin HK, Yamashita T, Ochiai K, Haller O, Watanabe T. Characterization and expression of the Mx1 gene in wild mouse species. Biochem Genet. 1998;36(9–10):311–22. doi: 10.1023/a:1018741312058 9919357

[55] Verhelst J, Spitaels J, Nürnberger C, De Vlieger D, Ysenbaert T, Staeheli P, et al. Functional comparison of Mx1 from two different mouse species reveals the involvement of loop L4 in the antiviral activity against influenza A viruses. J Virol. 2015;89(21):10879–90. doi: 10.1128/JVI.01744-15 26292322 PMC4621098

[56] Zeder MA. Domestication and early agriculture in the Mediterranean Basin: Origins, diffusion, and impact. Proc Natl Acad Sci U S A. 2008;105(33):11597–604. doi: 10.1073/pnas.0801317105 18697943 PMC2575338

[57] Marsden CD, Ortega-Del Vecchyo D, O’Brien DP, Taylor JF, Ramirez O, Vilà C, et al. Bottlenecks and selective sweeps during domestication have increased deleterious genetic variation in dogs. Proc Natl Acad Sci U S A. 2016;113(1):152–7. doi: 10.1073/pnas.1512501113 26699508 PMC4711855

[58] Ponten A, Sick C, Weeber M, Haller O, Kochs G. Dominant-negative mutants of human MxA protein: domains in the carboxy-terminal moiety are important for oligomerization and antiviral activity. J Virol. 1997;71(4):2591–9. doi: 10.1128/JVI.71.4.2591-2599.1997 9060610 PMC191379

[59] Chen Y, Graf L, Chen T, Liao Q, Bai T, Petric PP, et al. Rare variant MX1 alleles increase human susceptibility to zoonotic H7N9 influenza virus. Science. 2021;373(6557):918–22. doi: 10.1126/science.abg5953 34413236

[60] Forster J, Schweizer M, Schumacher RF, Kaufmehl K, Lob S. MxA protein in infants and children with respiratory tract infection. Acta Paediatr. 1996;85(2):163–7. doi: 10.1111/j.1651-2227.1996.tb13985.x 8640043

[61] Halminen M, Ilonen J, Julkunen I, Ruuskanen O, Simell O, Mäkelä MJ. Expression of MxA protein in blood lymphocytes discriminates between viral and bacterial infections in febrile children. Pediatr Res. 1997;41(5):647–50. doi: 10.1203/00006450-199705000-00008 9128286

[62] Tsao Y-T, Tsai Y-H, Liao W-T, Shen C-J, Shen C-F, Cheng C-M. Differential Markers of Bacterial and Viral Infections in Children for Point-of-Care Testing. Trends Mol Med. 2020;26(12):1118–32. doi: 10.1016/j.molmed.2020.09.004 33008730 PMC7522093

[63] Caserta LC, Frye EA, Butt SL, Laverack M, Nooruzzaman M, Covaleda LM, et al. Spillover of highly pathogenic avian influenza H5N1 virus to dairy cattle. Nature. 2024;634(8034):669–76. doi: 10.1038/s41586-024-07849-4 39053575 PMC11485258

[64] Hunt PW, Kijas J, Ingham A. Understanding parasitic infection in sheep to design more efficient animal selection strategies. Vet J. 2013;197(2):143–52. doi: 10.1016/j.tvjl.2013.03.029 23680266

[65] Hunt P, Ingham A. Parasite resistance in sheep - sheepgenomics program trials. v1. [Internet]. CSIRO Data Collection; 2023. Available from: doi: 10.25919/31xq-0p06

[66] Li H. A statistical framework for SNP calling, mutation discovery, association mapping and population genetical parameter estimation from sequencing data. Bioinformatics. 2011;27(21):2987–93. doi: 10.1093/bioinformatics/btr509 21903627 PMC3198575

[67] Kearse M, Moir R, Wilson A, Stones-Havas S, Cheung M, Sturrock S, et al. Geneious Basic: an integrated and extendable desktop software platform for the organization and analysis of sequence data. Bioinformatics. 2012;28(12):1647–9. doi: 10.1093/bioinformatics/bts199 22543367 PMC3371832

[68] Lee CE, Fulcher DA, Whittle B, Chand R, Fewings N, Field M, et al. Autosomal-dominant B-cell deficiency with alopecia due to a mutation in NFKB2 that results in nonprocessable p100. Blood. 2014;124(19):2964–72. doi: 10.1182/blood-2014-06-578542 25237204 PMC4321335

[69] François M, Karpe AV, Liu J-W, Beale DJ, Hor M, Hecker J, et al. Multi-Omics, an Integrated Approach to Identify Novel Blood Biomarkers of Alzheimer’s Disease. Metabolites. 2022;12(10):949. doi: 10.3390/metabo12100949 36295851 PMC9610280

[70] François M, Karpe A, Liu J-W, Beale D, Hor M, Hecker J, et al. Salivaomics as a Potential Tool for Predicting Alzheimer’s Disease During the Early Stages of Neurodegeneration. J Alzheimers Dis. 2021;82(3):1301–13. doi: 10.3233/JAD-210283 34151801 PMC8461673

[71] Ahmed KA, Yeap HL, Coppin CW, Liu J-W, Pandey G, Taylor PW, et al. Seminal fluid proteins in the Queensland fruit fly: Tissue origins, effects of mating and comparative genomics. Insect Biochem Mol Biol. 2025;177:104247. doi: 10.1016/j.ibmb.2024.104247 39667437

[72] Cox J, Mann M. MaxQuant enables high peptide identification rates, individualized p.p.b.-range mass accuracies and proteome-wide protein quantification. Nat Biotechnol. 2008;26(12):1367–72. 19029910 10.1038/nbt.1511

