# Supplementary material for: Identification of single nucleotide polymorphisms in sheep Mx genes: A premature stop codon abolishes Mx2 protein expression but did not affect fertility and early animal development
Source: PLoS One. 2026 Feb 11;21(2):e0337457. doi: 10.1371/journal.pone.0337457 (PMC12893586; doi:10.1371/journal.pone.0337457)
Supplement: S1 Table — (PDF) [file pone.0337457.s001.pdf]

Suppl. Table S1. — Impact predictions of amino acid substitutions in ovine Mx protein variants.

Gene	Pos.[a]	Codon	Change	SIFT [b]	PolyPhen-2 [b]	Protein identifier [c]
MX1	17	Gly	Ser	**Tolerated** (0.63)<br>Median sequence conservation: 4.32<br>Sequences no: 12	**Benign** (0.00)<br>Sensitivity: 1.00<br>Specificity: 0.00	NP_0010097531
	30	Met	Thr	**Tolerated** (0.41)<br>Median sequence conservation: 3.39<br>Sequences no: 20	**Benign** (0.00)<br>Sensitivity: 1.00<br>Specificity: 0.00	W5PKR2
	452	Ile	Val	**Tolerated** (0.13)<br>Median sequence conservation: 3.09<br>Sequence no.: 37	**Possibly damaging** (0.67)<br>Sensitivity: 0.86<br>Specificity: 0.91	NP_001072120.1
MX2	1	Met	Val	**Deleterious** (0.00) [g]<br>Median sequence conservation: 4.32<br>Sequence no.: 4	**Benign** (0.27)<br>Sensitivity: 0.91<br>Specificity: 0.88	NP_001072120.1
	64	Asn	His	**Tolerated** (0.13)<br>Median sequence conservation: 3.56<br>Sequence no: 6	**Possibly damaging** (0.58)<br>Sensitivity: 0.88<br>Specificity: 0.91	W5PKK1
	85	Pro	Ser	**Tolerated** (0.39)<br>Median sequence conservation: 3.32<br>Sequences no: 19	**Benign** (0.21)<br>Sensitivity: 0.92<br>Specificity: 0.88	W5PKK1
	713	Gly	Ser	**Deleterious** (0.00)<br>Median sequence conservation: 4.32<br>Sequence no.: 1	**Benign** (0.21)<br>Sensitivity: 0.92<br>Specificity: 0.88	W5PKK1

[a] Pos., amino acid position. [b] The impact of amino acid changes on protein function was analysed using two web-based bioinformatics tools: Sorting Intolerant from Tolerant (SIFT) (Ngak-Leng et al., 2012) and Polymorphism Phenotyping (PolyPhen) v.2 (Adzhubei et al., 2010). [c] Protein identifiers were used for query sequence. [d] A SIFT score of 0.05–1 indicates a “tolerated” substitution, whereas a score equal to or lower than 0.05 indicates a “deleterious” substitution. The median sequence conservation score and the sequence number (no.) specify

how conserved a residue is across species and where it occurs in the linear amino acid sequence, respectively. [e] A PolyPhen-2 score of 0.0–0.15 indicates a “benign” substitution, 0.15–0.85 indicates a “possibly damaging” substitution, and 0.85–1.0 indicates a “probably damaging” substitution. [e] This prediction was made with a low confidence, because the sequences used were not diverse enough. For a critical evaluation of how accurately SIFT and PolyPhen-2 predict a loss of protein function, see Flanagan et al. (2010).

References

Sim N-L, Kumar P, Hu J, Henikoff S, Schneider G, Ng PC. SIFT web server: predicting effects of amino acid substitutions on proteins. Nucleic Acids Res. 2012;40(1): 452–457.

Adzhubei IA, Schmidt S, Peshkin L, Ramensky VE, Gerasimova A, Bork P, et al. A method and server for predicting damaging missense mutations. Nat Methods. 2010;7(4): 248–249.

Flanagan SE, Patch AM, Ellard S. Using SIFT and PolyPhen to predict loss-of-function and gain-of-function mutations. Genet Test Mol Biomarkers. 2010;14(4): 533–537.
