# Supplementary material for: Identification of single nucleotide polymorphisms in sheep Mx genes: A premature stop codon abolishes Mx2 protein expression but did not affect fertility and early animal development
Source: PLoS One. 2026 Feb 11;21(2):e0337457. doi: 10.1371/journal.pone.0337457 (PMC12893586; doi:10.1371/journal.pone.0337457)
Supplement: S2 Table — (PDF) [file pone.0337457.s002.pdf]

Suppl. Table S2. — Birth weight and weight gain of lambs with functional and nonfunctional MX2 alleles.

Lamb ID	Date of birth	Sex	Weight at birth[a] (kg)	Weight at marking[a] (kg)	Age at marking (days)	Weight gain[b] (kg/day)	Lamb genotype[c] (W166*)	Dam ID	Dam genotype[c] (W166*)	Sire ID	Sire genotype[c] (W166*)
2018C8248	30/10/2018	M	3.99	12.5	30	0.284	-/-	2015C1656	-/-	2012A0020	-/-
2018C1508	27/10/2018	F	1.33	10.5	33	0.278	-/-	2016C1533	-/-	2012A0020	-/-
2018C1505	25/10/2018	M	3.35	13.5	35	0.290	-/-	2015C1533	-/-	2012A0020	-/-
2018C1512	1/11/2018	M	3.93	11.5	28	0.270	-/-	2015C1531	-/-	2012A0020	-/-
2018C1513	4/11/2018	M	1.74	5	25	0.130	-/-	2015C1532	-/-	2012A0020	-/-
2018C1514	4/11/2018	M	1.73	5.5	25	0.151	-/-	2015C1532	-/-	2012A0020	-/-
2018C1504	22/10/2018	M	3.63	14	38	0.273	-/-	2016C1571	+/-	2012A0020	-/-
2018C8251	2/11/2018	F	4.26	12	27	0.287	-/-	2015C1634	+/-	2012A0020	-/-
2018C1515	9/11/2018	M	3.83	7.5	20	0.184	-/-	2014C1531	+/-	2012A0020	-/-
2018C1510	29/10/2018	M	3.99	12	31	0.258	-/-	2015C1545	+/-	2012A0020	-/-
2018C1503	21/10/2018	F	2.6	9.5	39	0.177	-/-	2014C1536	+/-	2012A0020	-/-
2018C1516[d]	13/11/2018	F	2.9	–	16	–	-/-	2014C1532	+/-	2012A0020	-/-
2018C1501	20/10/2018	M	3	12	40	0.225	-/-	2015C1526	+/-	2012A0020	-/-
2018C1511	29/10/2018	F	3.71	10.5	31	0.219	-/-	2015C1524	+/-	2012A0020	-/-
2018C8246	25/10/2018	M	3.92	13.5	35	0.274	+/-	2015C1657	+/-	2012A0020	-/-
2018C1502	21/10/2018	M	3.3	11	39	0.197	+/-	2014C1536	+/-	2012A0020	-/-

2018C1506	27/10/2018	F	3.01	9.5	33	0.197	+/-	2014C1537	+/-	2012A0020	-/-
2018C1507	27/10/2018	F	2	7.5	33	0.167	+/-	2014C1537	+/-	2012A0020	-/-
2018C8243	24/10/2018	M	3.9	13.5	36	0.267	+/-	2016C1645	+/-	2012A0063	+/-
2018C8256	8/11/2018	F	3.27	8.5	21	0.249	+/-	2016C1597	+/-	2012A0063	+/-
2018C1509	28/10/2018	M	3.87	11.5	32	0.238	+/-	2016C1551	+/+	2012A0020	-/-
2018C8255	3/11/2018	F	2.68	9	26	0.243	+/-	2016C1655	+/+	2012A0063	+/-
2018C8242	21/10/2018	F	2.54	12	39	0.243	+/-	2016C1652	+/+	2012A0063	+/-
2018C8250	1/11/2018	M	4.07	12	28	0.283	+/-	2016C1646	+/+	2012A0063	+/-
2018C8252	3/11/2018	M	3.49	10	26	0.250	+/+	2016C1614	+/+	2012A0063	+/-
2018C8244	24/10/2018	M	4.12	13	36	0.247	+/+	2016C1633	+/+	2012A0063	+/-
2018C8247	25/10/2018	F	3.61	12.5	35	0.254	+/+	2016C1617	+/+	2012A0063	+/-
2018C8254	3/11/2018	F	3.14	9.5	26	0.245	+/+	2016C1653	+/+	2012A0063	+/-
2018C8249	31/10/2018	M	2.89	9.5	29	0.228	+/+	2016C1600	+/+	2012A0063	+/-
2018C8253	3/11/2018	M	3.78	11	26	0.278	+/+	2016C1634	+/+	2012A0063	+/-
2018C8258[d]	19/11/2018	M	3.6	–	10	–	+/+	2015C1633	+/+	2012A0063	+/-

[a] Lambs were weighed at birth and when they were tail-docked and males were castrated (marking). [b] Weight gain was calculated for the time between birth and marking. [c] The presence of a SNP (W166*) that prematurely terminates the Mx2 ORF prematurely was analysed using PCR. The three variates "Weight at birth", "Weight at marking", and "Weight gain" were analysed using ANOVA (unbalanced ANOVA) in Genstat (24th edition). None of these variates differed significantly between in *MX2* positive (+/+ and +/-) and *MX2* negative (-/-) lambs, dams, or sires ("Weight at birth": p = 0.592, p = 0.116, p = 0.369 for lambs, dams, and sires, respectively; "Weight at marking": p = 0.909, p = 0.559, p = 0.617 for lambs, dams and sires, respectively; "Weight

gain": $p = 0.724$, $p = 0.482$, $p = 0.126$ for lambs, dams and sires, respectively). Animals 2018C8258 and 2018C1516 were excluded from the statistical analysis of weight gain and marking weight because of their late birth dates.
