# Supplementary material for: Identification of single nucleotide polymorphisms in sheep Mx genes: A premature stop codon abolishes Mx2 protein expression but did not affect fertility and early animal development
Source: PLoS One. 2026 Feb 11;21(2):e0337457. doi: 10.1371/journal.pone.0337457 (PMC12893586; doi:10.1371/journal.pone.0337457)
Supplement: S3 Table — (PDF) [file pone.0337457.s003.pdf]

Suppl. Table S3. — Identification of Mx1 peptides by LC-MS.

Pos.[a] (in Mx1)	Amino acid sequence[b]	No. of peptides[c]			*m/z*	Mass (Da)	Score[d]
		+/+	+/ W166*	W166*/ W166*			
2–26	VLSDLDIKEPDSPESGLNGSDDMVR	2	2	3	897	2,687	185
119–36	VSFLDREIEISDASQVEK	2	0	2	690	2,064	84
125–36	EIEISDASQVEK	2	0	1	674	1,347	116
137–79	EISEAQIAIAGEGMGISHELISLEVSSPHVPD LTLIDLPGITR	1	1	0	1,128	4,507	65
180–94	VAVGNQPHDIEYQIK	4	3	5	571	1,710	207
204–25	QETINLVVVPANVDIATTEALR	1	1	1	1,184	2,365	61
226–36	MAQDVDPQGDR	3	2	1	616	1,231	96
296–305	IFFEDHTHFR	3	5	3	450	1,348	156
333–42	TLPLLENQIK	2	2	4	585	1,168	84
34-43	GNLYSQYEEK	1	2	1	616	1,230	142
355–66	YGKDIPEEESEK	2	1	1	475	1,423	89
367–73	MFSLIEK	2	2	1	434	867	84
409–15	WSAVVEK	1	1	1	410	817	83
437–47	GRELPGFVNYK	1	0	0	427	1,279	103
439–47	ELPGFVNYK	3	0	2	534	1,066	88
477–85	NTFTEVSGK	1	1	1	492	982	80
486–95	HFSEFFNLHR	5	2	4	445	1,333	155
499–514	SKIEDIRLEQENEAEK	2	0	2	644	1,930	144
553–83	SNHYYQSEDSEPSTAEIFQHLMAYHQEVST R	6	5	4	738	3,684	130
607–23	SMLQLLQDKDQYDWLLK	8	6	7	713	2,136	202

[a] Position of peptide. [b] Peptide sequence. [c] Number of peptides detected in wild-type fibroblasts with a functional *MX2* allele (+/+) and fibroblasts heterozygous or homozygous for a SNP (W166*) that terminates the ORF

prematurely. All cells were treated with 100 IU IFN-α for 24 hours before cell lysates were subjected to mass spectrometry. [d] Probability score calculated as -10 $\log_{10}$ (p value).
