# Supplementary material for: Identification of single nucleotide polymorphisms in sheep Mx genes: A premature stop codon abolishes Mx2 protein expression but did not affect fertility and early animal development
Source: PLoS One. 2026 Feb 11;21(2):e0337457. doi: 10.1371/journal.pone.0337457 (PMC12893586; doi:10.1371/journal.pone.0337457)
Supplement: S4 Table — (PDF) [file pone.0337457.s004.pdf]

Suppl. Table S4. — Identification of Mx2 peptides by LC-MS.

Pos.[a] (in Mx2)	Amino acid sequence[b]	No. of peptides[c]			*m/z*	Mass (Da)	Score[d]
		+/+	+/ W166*	W166*/ W166*			
12–23	RHAPTSTQHHPK	1	0	0	466	1,396	88
13–23	HAPTSTQHHPK	2	2	0	621	1,240	129
174–87	NVTQQLHNPSEVER	3	1	0	826	1,650	214
192–230	AQNIIAGNGVGISHELINLEVTSPEVPDLTLI DLPGITR	3	0	0	1,360	4,078	51
231–45	VAVENQPQDIGLQIK	7	3	0	827	1,651	203
336–43	LSLAEATR	1	0	0	431	860	68
345–56	EVMFFQTHPYFR	3	2	0	535	1,601	153
373–83	LTTELIWHINK	5	1	0	684	1,367	118
399–417	ATEELQQYGDDIPSNEGDK	6	0	0	1,055	2,108	392
399–424	ATEELQQYGDDIPSNEGDKMFFLIEK	1	2	0	1,007	3,016	139
418–24	MFFLIEK	3	1	0	464	927	101
473–85	SILNEEVSKYETK	2	0	0	514	1,539	70
591–601	VREEIFNSVGK	1	0	0	427	1,277	107
640–59	LANQIPFIIQYFMLQENGDK	3	2	0	795	2,381	168
640–62	LANQIPFIIQYFMLQENGDKVQK	2	0	0	913	2,736	99
663–87	AMMQLLQETQHYSWLLQEQSDTATK	7	2	0	994	2,979	201
663–88	AMMQLLQETQHYSWLLQEQSDTATKR	2	0	0	785	3,136	129
698–712	LTQAQQALYEFPHFK	2	1	0	608	1,820	181
698–712	LTQAQQALYEFPHFKG	4	1	0	627	1,877	126

[a] Position of peptide. [b] Peptide sequence. [c] Number of peptides detected in wild-type fibroblasts with a functional *MX2* allele (+/+) and fibroblasts heterozygous or homozygous for a SNP (W166*) that terminates the ORF prematurely. All cells were treated with 100 IU IFN-α for 24 hours before cell lysates were subjected to mass spectrometry. [d] Probability score calculated as -10 $\log_{10}$ (p value).
