# Supplementary material for: Identification of single nucleotide polymorphisms in sheep Mx genes: A premature stop codon abolishes Mx2 protein expression but did not affect fertility and early animal development
Source: PLoS One. 2026 Feb 11;21(2):e0337457. doi: 10.1371/journal.pone.0337457 (PMC12893586; doi:10.1371/journal.pone.0337457)
Supplement: S5 Table — (PDF) [file pone.0337457.s005.pdf]

Suppl. Table S5. — Identification of Mx1/Mx2 peptides by LC-MS.

Pos.[a] (in Mx1)	Pos.[a] (in Mx2)	Amino acid sequence[b]	No. of peptides[c]			*m/z*	Mass (Da)	Score[d]
			+/+	+/ W166*	W166*/ W166*			
56–78	109–31	ALGVEQDLALPAIAVIGDQSSGK	22	16	19	1,127	2,251	311
79–92	132–45	SSVLEALSGVALPR	20	16	11	467	1,398	230
237–49	288–300	TIGILTKPDLVDK	6	3	2	472	1,412	148

[a] Position of peptide. [b] Peptide sequence. [c] Number of peptides detected in wild-type fibroblasts with a functional *MX2* allele (+/+) and fibroblasts heterozygous or homozygous for a SNP (W166*) that terminates the ORF prematurely. All cells were treated with 100 IU IFN-α for 24 hours before cell lysates were subjected to mass spectrometry. [d] Probability score calculated as -10 $\log_{10}$ (*P* value).
