# Supplementary material for: Identification of single nucleotide polymorphisms in sheep Mx genes: A premature stop codon abolishes Mx2 protein expression but did not affect fertility and early animal development
Source: PLoS One. 2026 Feb 11;21(2):e0337457. doi: 10.1371/journal.pone.0337457 (PMC12893586; doi:10.1371/journal.pone.0337457)
Supplement: S1 Fig — (PDF) [file pone.0337457.s006.pdf]

Suppl. Fig S1. – Regulatory elements in ovine Mx gene promoters.

A

***MX1* promoter region**

```
                                    SP1     NF-kB
TTCTTAGCTG AGCCAAGGAG GATGGAGGTG GGCTTTCCAA AATGAAATTT TACTCCTCAT
                              GC Box          ISRE

CCCCAAAGCC CTCCGGGAGG GCTCATCTTT GGGTCTATTT GACCTTGGTC CCCCACTTTC

                                                                NF-kB
TACTCATTTC CATCCTGAAG AAATCAAATG AGCTAAACTT CAGGGAGGGC TTGGGACTTC
                 CEBPB                             GC Box

CCTGGTGGTC CAGGGAACCT TTTGCTTCCA ATTCAGGGGG CACAGGTCCG ATCCCTGGTC

AAGAAACCAA GATCTCACAC GACATGCAGA GTGGTCAAGA GATTTAAAGG TGGGTGGAGG

   SP1                         CEBPB
GGGCGGTGCT TGAAGAGGAT AAGGAAAATT GAGAAAGGAG CTTGATGTAG GTCTGGGTGA
   GC Box           ISRE

GGAGGGGGCT AAGGGGGTAG CTCCCTAGCA TGCTTGAGAA TCCCTACGGG CGCTGGACTG
  GC Box

TTCCCAGTGA ACCTACAGCA GAAGTTTGAT ACCCAATTAT CAATGCATCT GTTCAAACAA

CCAAAGGTTA AGGTTAGCTA GGTTCCAAGC TACCTTCGGC TTTGGATGAC TCGGGGCTGC
                                                        AP1

TTGAGCAGAG GTTCTCAAAC TGCCAGAAAC TTCAGAAGGG CCGGATAAAG CTCGGGTAGC

TGGGTCCTGC TCCCGAGTTT CTGGGGCAGC AGGTCTGGGT GCGGGCCGAG AATTTGCATT
                                                             ISRE

TCCCGCAAGC TCCTAGGGAT GCTGTTGGTG CGGGGCGCAC CTAGAGTGCT TCTGGGAGGA

TACAGCTGAG GGTGCTGGGC GCAGCGACCT CGGGAGGCGC CGGTGCGCAA GTGCGCTACC

            ISRE        ISRE
CGTTCGATTT GGGTTTCGGT TTCCTTTCCG ATTCAGCAGC CCTGAAAACT CTACGAGTTT
                 ISRE       ISRE
                              SP1
CGTTTCCCAG AGGCTGGGTG GGAGATGACG GACGGGGAGG CGGGGGCAGC GAGCTGGGGG
  ISRE        SP1                          GC Box          GC Box

CGGCGCTAGC GCTGCATAAA GCCGAGGAGG GCCAGCGCCG GGAGCCCGCC GGTCACTGCG

CTGGGGACGG GTAAGTGTAG CGCTCCTGGG TCTGGGCACC CTCCGCGGGT CGGGGCATTT
```

B

***MX2* promoter region**

TGGATGGATG ACCTGGGCAT CAGATCCTGC AGGACGCAGA CCCTGGAAAC TACCCCCAGG
SP1

ATCTCTTAAT GCCAGGCTTT AATAACCATC TTTTAAGCCT AAAAATGTCT TCTTTAGAGG

AAATTTTGCC CACAAGTCAG GTGAATGAGC AAAAAAGTGC TCCGGATTAA TTTTGTTAAT
CEBPB CEBPB

TTTCTTAGAT TTTTTGTTTT GTTTTGGAAA GCTTCATGGG TGTGACGCTG AGATGGAGGC
CEBPB GC Box

SP1
AGAATCCCCC CGTCCTGTGC CAGCCCTGAA CCCCCAGGGC TCCTTGGGGC TCCCCATACT
SP1 NF-kB

SP1
CTGGTGGTGG GAATGCCCAG CTCACCCTCG CTTCAGTCAC AGCGAAGGGA GGGCTTTGGG
NF-kB GC Box

ACCCCAGGAG GTGCATGCCC ACAGTTTTGC AGGCGAAGTA ACCCACACAG GACCAGGAAG
SP1

TCAGGCTCAC CCCTGGGAAC TAGCTCATGT GTGAGGGTAT GCACCGCGTG CCTACGTGAG

GGATGCTGTG GAGGAGCTGT GGATGTCCCT TCCTAGCATA TTCAGAGCTT AAACAGGGCA
SP1

GAGAGTGGCA GCGCACAGCA TCTCAGAAAG TCCTTCTGGA CAGTGCTATG ATCAGTGACT

CGTTCTCCAA GTGTGCTCGG CCCCAGCCTG CTGGAGAACC TGTTAGCCCC GTTACCCGCC

CCCACCCCGC CCCTCCCTGT GAGTTCAAAT GCCCACCAGG AGACTCATGC AGCTGGAGTC
GC Box

AGGACCACTG TGTGAAGCAC CGGGAAATGG ATGGATGCTT CAGGTTTCGT TTCTGTGGCA

ISRE
GGCTGATGGT TTCGTTTCTA TGGCAAGCCA TTAGTTTCAT TTCCCTTGCT TCTGGGAAAG
ISRE

CAAGCCACGA GGGGAGGAGG GATCCTATAA AAGGAACGGG AGGAATGTGC AGAGAT**GACT**
SP1 GC Box TATA box CEBPB

TGCCGGCCTC CCTCCTACAG GCGCACAGGT AAGAGCGGGG TCAGTGCGGG GAGGGCTGCC

Suppl. Fig S1. Regulatory elements in ovine Mx gene promoters. (A, B) *MX1* and *MX2* promotor sequences were retrieved from NCBI genome datasets, i.e., access no. GCA_ 016772045.2 (Chr 1:262,347,688–8,705) and access no. GCA_000298735 (Chr 1:259,514,839–5,618), respectively. Approximately 1,000 nucleotides upstream of exon 1 were analysed to identify putative transcription factor binding sites and ISRE motif (5'-TTTCNNTTTC-3') using the TFBIND tool (https://tfbind.hgc.jp/; Tsunoda & Tagaki, 1999) and Geneious Prime (https:// www. geneious.com), respectively. For a conservative approach, analyses were conducted with cut-off values of 0.85 and 0.9 for transcription factor binding sites and the ISRE motif, respectively. Exon 1 sequences are printed in bold. AP1, activator protein 1; CEBPB, CCAAT/ enhancer-binding protein beta (also named nuclear factor interleukin 6); SP1, specificity protein 1; NF-kB, nuclear factor kappa-light-chain-enhancer; ISRE, interferon-stimulated response element.

References

Tsunoda T, Takagi T. 1999. Estimating transcription factor bindability on DNA. Bioinformatics. 1999;15(7–8): 622–630.
