# Supplementary material for: Identification of single nucleotide polymorphisms in sheep Mx genes: A premature stop codon abolishes Mx2 protein expression but did not affect fertility and early animal development
Source: PLoS One. 2026 Feb 11;21(2):e0337457. doi: 10.1371/journal.pone.0337457 (PMC12893586; doi:10.1371/journal.pone.0337457)
Supplement: S1 File — (PDF) [file pone.0337457.s007.pdf]

Supplementary Material S1 – Raw images of Western blots)

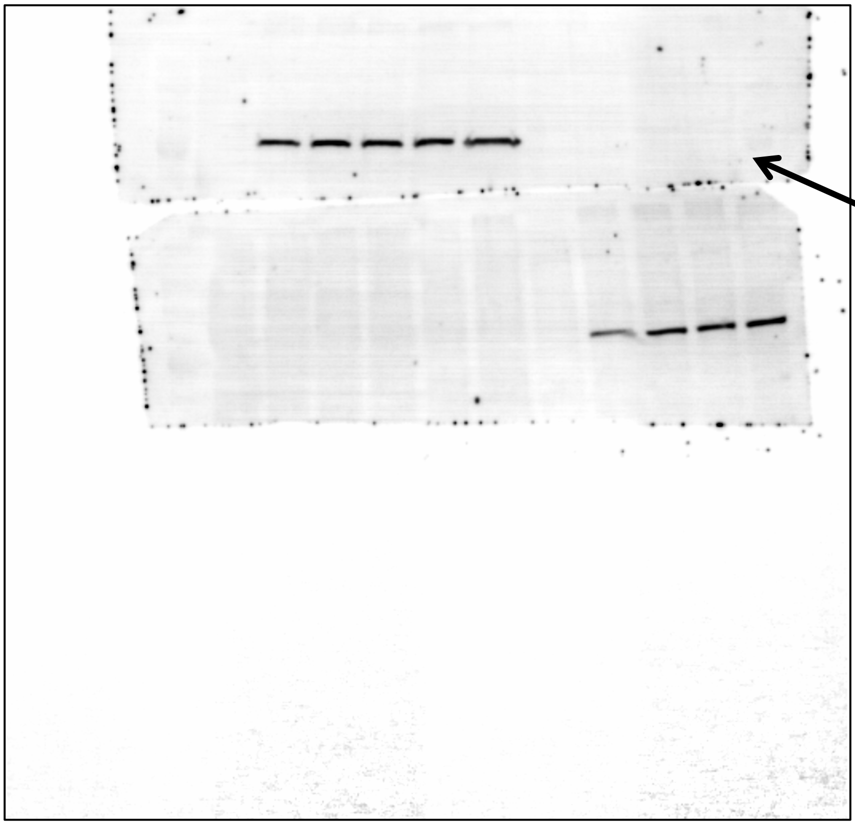

Blot stained for Mx2 (depicted in Fig 4B)

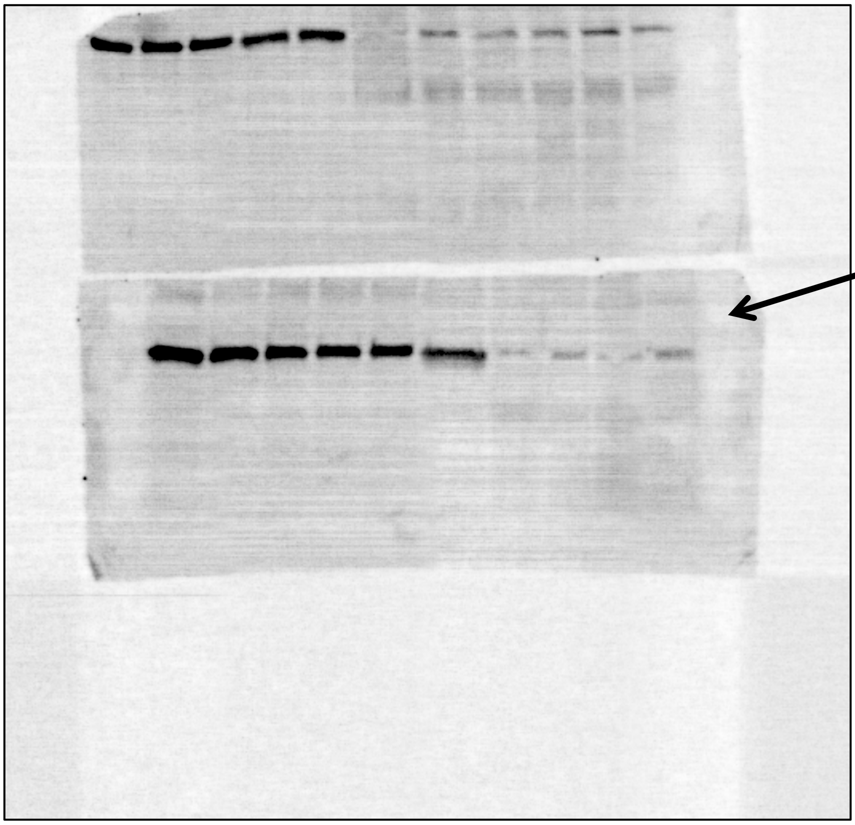

Blot stained for beta-actin (depicted in Fig 4B)

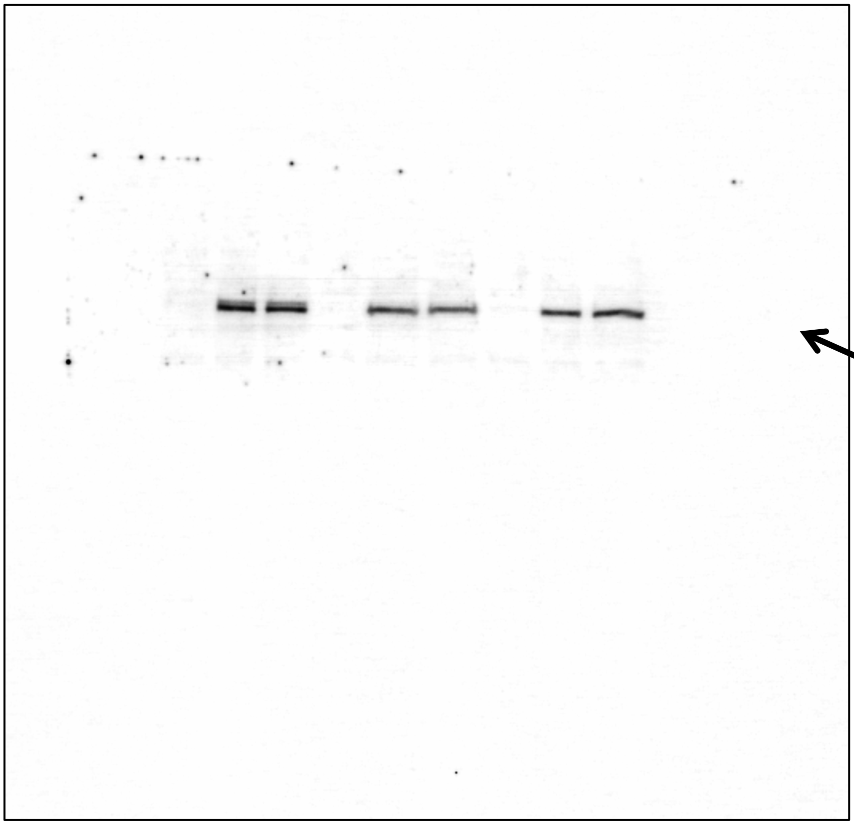

Blot stained for Mx1
(depicted in Fig 4D)

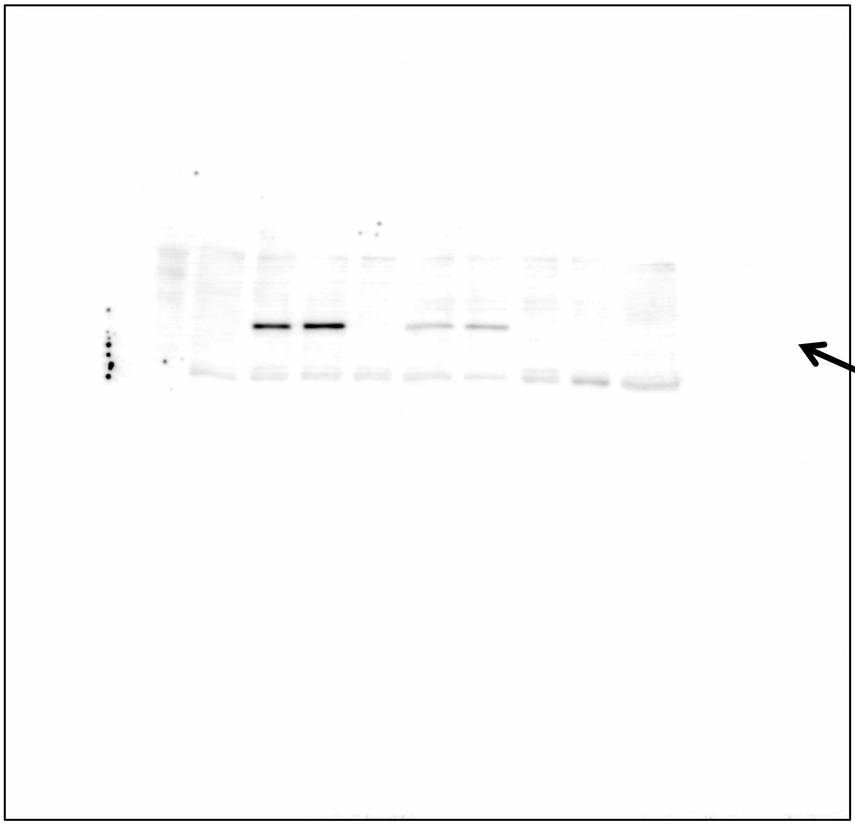

Blot stained for Mx2
(depicted in Fig 4D)

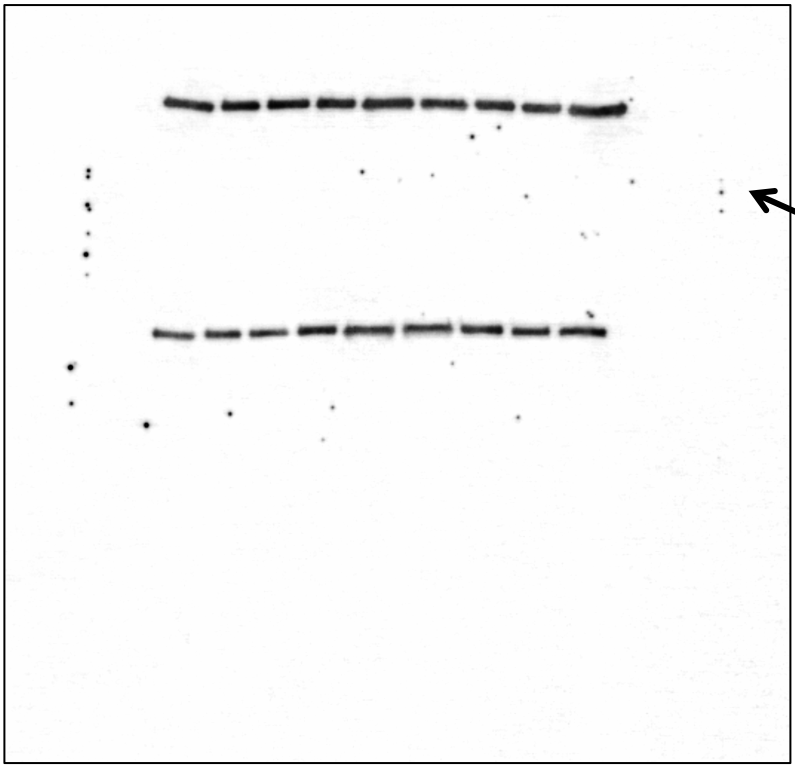

Blot stained for beta-actin (depicted in Fig 4D)
